# The impact of phage treatment on bacterial community structure is minor compared to antibiotics

**DOI:** 10.1038/s41598-023-48434-5

**Published:** 2023-11-29

**Authors:** Madeleine S. Gundersen, Alexander W. Fiedler, Ingrid Bakke, Olav Vadstein

**Affiliations:** Department of Biotechnology and Food Science, Norwegian University of Science and Technology (NTNU), Trondheim, Norway

**Keywords:** Community ecology, Microbial ecology

## Abstract

Phage treatment is suggested as an alternative to antibiotics; however, there is limited knowledge of how phage treatment impacts resident bacterial community structure. When phages induce bacterial lysis, resources become available to the resident community. Therefore, the density of the target bacterium is essential to consider when investigating the effect of phage treatment. This has never been studied. Thus, we invaded microcosms containing a lake-derived community with *Flavobacterium columnare* strain Fc7 at no, low or high densities, and treated them with either the bacteriophage FCL-2, the antibiotic Penicillin or kept them untreated (3 × 3 factorial design). The communities were sampled over the course of one week, and bacterial community composition and density were examined by 16S rDNA amplicon sequencing and flow cytometry. We show that phage treatment had minor impacts on the resident community when the host *F. columnare* Fc7 of the phage was present, as it caused no significant differences in bacterial density α- and β-diversity, successional patterns, and community assembly. However, a significant change was observed in community composition when the phage host was absent, mainly driven by a substantial increase in *Aquirufa*. In contrast, antibiotics induced significant changes in all community characteristics investigated. The most crucial finding was a bloom of *γ-proteobacteria* and a shift from selection to ecological drift dominating community assembly. This study investigated whether the amount of a bacterial host impacted the effect of phage treatment on community structure. We conclude that phage treatment did not significantly affect the diversity or composition of the bacterial communities when the phage host was present, but introduced changes when the host was absent. In contrast, antibiotic treatment was highly disturbing to community structure. Moreover, higher amounts of the bacterial host of the phage increased the contribution of stochastic community assembly and resulted in a feast-famine like response in bacterial density in all treatment groups. This finding emphasises that the invader density used in bacterial invasion studies impacts the experimental reproducibility. Overall, this study supports that phage treatment is substantially less disturbing to bacterial communities than antibiotic treatments.

Over the last seven decades, antibiotics have been the dominating method to treat bacterial infections^1^. However, their overuse and misuse have led to antibiotic-resistant bacteria at a fast rate^2^, and it is now evident that antibiotics can negatively impact the resident bacterial community^3^. As a result, it is critical to find alternatives to antibiotics to combat bacterial infections^4,5^. One potential alternative is phage therapy, which utilises viruses that specifically kill target bacteria^6^.

Antibiotics have numerous times been documented to affect the resident microbial community, leading to, for example, decreased bacterial growth, diversity, stability, and functionality and causing overall changes in community composition^3,7–9^. In many reports, antibiotic usage disrupts the community beyond its resilience, leaving lasting effects on the microbiome^7,10^. Changes in the competitive fitness of the populations^11^ and broken interaction networks^9^ might be underlying ecological mechanisms for the lasting disturbance effects. Because of the ever-growing evidence for the importance of the microbiome for ecosystem functioning, developing treatments that do not disturb the resident bacterial community is imperative^12,13^.

Phages are vital constituents of natural ecosystems^14,15^ and are critical for sustaining high productivity and ecosystem turnover^16^. The phages have a very narrow host specificity, even down to the strain level, and are self-propagating when the host is present^17^. Their therapeutic potential was explored in 1919 by d’Hérelle, just two years after he discovered bacteriophages, and is known as phage therapy^6^.

It is frequently stated that phage therapy does not disturb the resident bacterial community^18,19^. However, few studies have investigated the impact of phage therapy on the resident bacterial community, and conclusions vary. Most studies have been conducted in animals and humans without the presence of the bacterial host, and aimed to evaluate the safety of phage consumption^20–28^. Moreover, although phage therapy is proposed as an alternative to antibiotics, hardly any studies have compared the impact of phage- versus antibiotic treatment in communities containing the bacterial host of the phage^29–31^. In these studies, phage treatment induced changes in the bacterial communities of rabbits^29^, mice^30^ and a human-gut synthetic community^31^, but was less disruptive to the bacterial communities than antibiotics.

Furthermore, if phage treatment replaces antibiotics, it is critical to evaluate the impact of phage introduction on environmental ecosystems. To our knowledge, only two studies have addressed this issue, but neither had a relevant disturbance control. When phages were added to water, no significant changes were observed in the bacterial community^32^, while significant changes were observed when phages were added to soil^33^. Importantly, no studies have manipulated the density of the phage’s bacterial host.

From an ecological perspective, one would expect that the abundance of the host bacterium would influence the effects of the phage treatment on the resident community. This is because the lysis of the target bacterium releases mineral nutrients and dissolved organic material (DOM) that stimulate the growth of the resident community^34^. Overall, the impact of phage therapy on the resident microbiome vary and may depend on the population size of the host, and the mechanisms behind the effects are unclear.

In this study, we investigated if phage treatment and different amount of phage-host impacted bacterial community structure. We hypothesised that higher amounts of the bacterial host would release more resources due to bacterial lysis, leading to a greater impact of the treatments on the community composition. A bacterial community from the planktonic fraction of a natural lake ecosystem was added the bacterial host of the phage at two different densities or left uninvaded, and the added the phage. We used the broad-spectrum antibiotic Penicillin as a positive control for bacterial community disturbance. Both phage and antibiotic treatment cause bacterial lysis. We investigated the impact of phage and antibiotic treatment on community cell density, structure, diversity, and assembly and to evaluated if the amount of phage host modulates these effects. We analysed the bacterial community over a week using 16S rRNA gene amplicon sequencing and flow cytometry.

## Methods

### Experimental design

**Figure 1:**
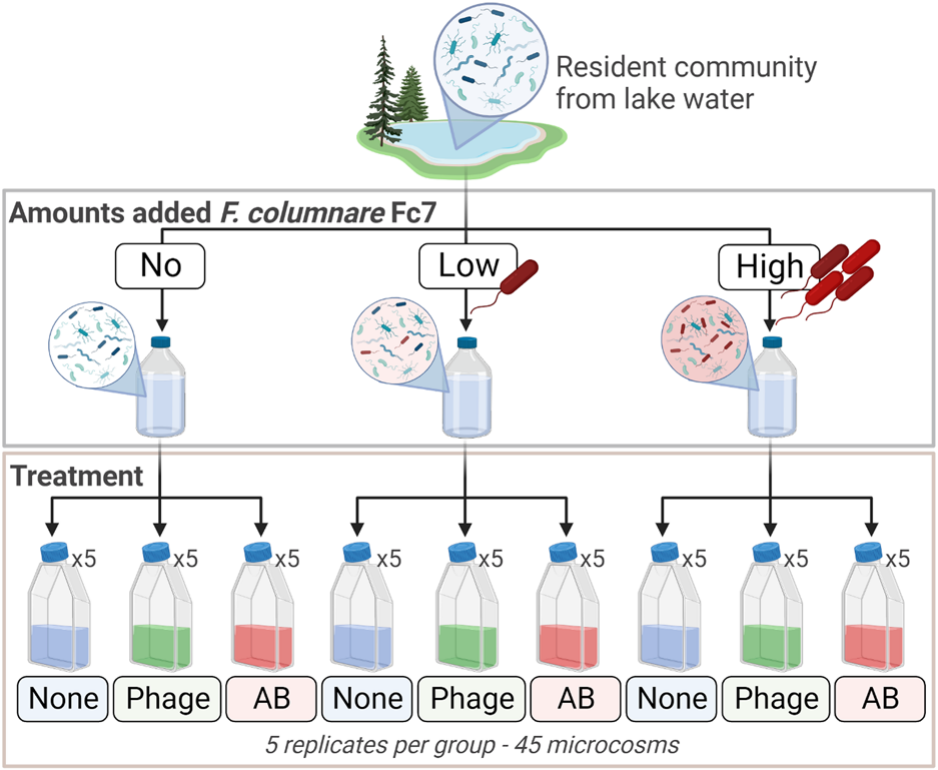
*Flavobacterium columnare* strain Fc7 was added to a lake bacterial community (i.e. the resident community) in three different amounts (no, low or high addition). After mixing the resident community and *F. columnare* Fc7, the communities were split into five experimental microcosms (100 mL each) and received treatment. The communities were treated with nothing (“none”), the phage FCL-2 (Phage) or the antibiotic Penicillin (AB). The experiment consisted of nine groups replicated five times resulting in a total of 45 microcosms.

A 3 × 3 factorial experiment was conducted to compare the effect of phage- and antibiotic treatment on resident bacterial community properties after an invasion by *Flavobacterium columnare* strain Fc7 (Fig. 1). We varied the amount of the invader (no, low and high) and the treatment type (phage, antibiotic or none) to obtain nine experimental groups. Each experimental group comprised five replicates yielding a total of 45 microcosms (250 mL cell culture flasks with ventilated caps). The experiment was conducted at 14 °C. The microcosms were invaded at day 0, and treatments were applied one hour after the invasion. Phage- and antibiotic treatment was applied once by adding phage FCL-2 to a multiplicity of infection (MOI) of 2.3 (low) and 2.9 (high) or adding 1 mg/L of the antibiotic Penicillin. To secure community turnover, we exchanged 11% of the microcosm daily with 0.2 µm-filtered, autoclaved lake water, keeping the volume constant at 100 mL. The experiment was terminated after 7 days.

### *Flavobacterium columnare* strain Fc7

The Gram-negative freshwater bacterium *Flavobacterium columnare* strain Fc7 was used as the invader^35^. *F. columnare* Fc7 was cultivated in TYES medium at room temperature (22 °C) under aerobic conditions and with shaking^35^. *F. columnare* Fc7 was taken from a glycerol stock three days before starting the experiment, and 5% v/v was transferred daily in liquid TYES to keep the culture in the exponential phase. One mL of an *F. columnare* Fc7 culture in the late exponential phase was harvested by centrifugation (13,000*g*, 5 min) and resuspended in 1 mL 0.2 µm filtered lake water. The density of the harvested *F. columnare* Fc7 was quantified to be 3.4 × 10^8^ cells/mL using flow cytometry.

### Resident bacterial community

The resident bacterial community was collected at 50 m depth from a lake (Jonsvatnet, Trondheim, Norway) in May 2022. The collected water was filtered with a 55 µm screen to remove larger protozoa and had a bacterial density of 6.25 × 10^5^ cells/mL. The filtered lake water was split into three 2L bottles. *F. columnare* Fc7 was introduced to two of these bottles by adding low (1.38 × 10^5^ *F. columnare* Fc7 cells/mL. 24% bacterial density increase) or high amounts of *F. columnare* Fc7 (1.07 × 10^6^ *F. columnare* Fc7 cells/mL, 190% bacterial density increase = high) at day 0. The bottles were shaken well, and 100 mL was transferred to each microcosm before the treatments were applied.

### Phage treatment with FCL-2

The phage FCL-2, which targets *F. columnare* Fc7, was used for the phage treatment^36^. Selectivity towards *F. columnare* Fc7 was confirmed using the soft-agar overlay technique and spot testing^37^. We prepared an FCL-2 phage stock containing 10^10^ PFU/mL. More details on phage stock preparation are given in Supplementary methods. We added 3.12 × 10^5^ PFU/mL to the microcosms with low amounts of *F. columnare* Fc7 added and 3.12 × 10^6^ PFU/mL to the microcosms added high amounts of *F. columnare* Fc7 to obtain an MOI of 2.3 and 2.9, respectively. 3.12 × 10^6^ PFU/mL were also added to the microcosms without *F. columnare* Fc7 added to account for the impact of phages when no host is present.

### Estimation of total and living bacterial community density

Each day before the water exchange, 1 mL from each microcosm was fixed with glutaraldehyde (0.1% final), snap-frozen and stored at − 80 °C (45 samples/day). We sampled an additional 1 mL from replicate microcosm #3 for immediate live-dead cell density analysis (9 samples/day). The bacterial density was quantified using flow cytometry (Attune NxT, ThermoFisher). Fixed samples were stained with the RNA-binding fluorescent stain SYBR green II (Invitrogen) to quantify the total bacterial density. To quantify the living population, we immediately stained samples with two dyes; the fluorescent DNA-binding dyes SYBR green I (Invitrogen) and propidium iodide (PI), which enter all or only membrane-compromised cells, respectively. An in-depth description of the protocol, system configurations and gating strategy is given in the Supplementary methods.

### Sampling for bacterial community characterisation

At day 1, 3 and 7, 10 mL of the water from each microcosm was filtered through a 0.2 µm polycarbonate filter (Osmonics, 25 mm) to sample the bacterial community. In addition, two technical replicates were sampled from the original lake water before the invasion and two technical replicates from each 2L flask with varying invader density (3 groups). The filters were placed in 1.5 mL cryo tubes, snap-frozen and stored at -80 °C until DNA extraction.

### DNA extraction

For extraction of bacterial DNA, each filter was cut into pieces and homogenised in 750µL DNA/RNA Shield solution (Zymo Research) in ZR BashingBead Lysis Tubes (0.1- and 0.5-mm matrix) using a Precellys 24 (5500 rpm-2 × 30 s-15 s break, Bertin Technologies). Next, DNA was extracted and purified using the ZymoBiomics MagBead DNA/RNA kit (R2135, Zymo Research) and the KingFisher Flex automated extraction instrument according to the manufacturer’s protocol, except for eluting DNA in 100 µL water instead of 50 µL. Extracted DNA was stored at − 20 °C.

### 16S rRNA gene amplicon sequencing and processing

The V3-V4 region of the 16S rRNA gene was amplified using the broad-coverage PCR primers Ill341F-KL (5′-TCG-TCG-GCA-GCG-TCA-GAT-GTG-TAT-AAG-AGA-CAG-NNN-NCC-TAC-GGG-N-3′) and Ill805R (5′-GTC-TCG-TGG-GCT-CGG-AGA-TGT-GTA-TAA-GAG-ACA-GNN-NNG-ACT-CAN-VGG-GTA-TCT-AAK-CC-3′). Each reaction was run for 36 cycles (98 °C 15 s, 55 °C 20 s, 72 °C 20 s) with final concentrations of 0.15 μM of each primer, 0.25 mM of each dNTP, 1 × Phusion buffer HF, 0.015 units/μL of Phusion Hot Start II DNA polymerase and 1μL of DNA extracts as the template in 25 μL reaction volume. PCR products were examined using electrophoresis on 1% agarose gels (1 h, 110 V) containing 50 µM GelRed (Biotium). The amplicon library was prepared as described previously^38^ by first purifying and normalising the amplicons using the SequalPrepTM Normalization Plate Kit (Invitrogen) before samples were dual-indexed with Illumina adapters using PCR (FC-131-2001 and 2003, 10 cycles). The indexed amplicons were normalised and purified again before the library was concentrated using Amicon® Ultra-0.5 Centrifugal Filter Devices. The library was sequenced using MiSeq v3 Illumina sequencing (Illumina, San Diego, CA) employing 300 base pair paired reads at the Norwegian Sequencing Centre at the University of Oslo, Norway. The Illumina sequencing reads were processed using the USEARCH pipeline^39^ (v.11). An amplicon sequence variant (ASV) table was generated as described previously^40^, except for removing reads < 370 base pairs instead of 400.

### Statistical analysis

All data analysis was performed in R^41^ (v. 4.2.2.) with 3003 as a seed. Quality assurance of the amplicon data and normalising strategy can be found in the Supplementary methods. All R-scripts are available at github.com/madeleine-gundersen/Phage_impact_community. α-diversity was investigated as Hill diversity of order 0 (richness), 1 and 2^42^ using the normalised amplicon library. For bacterial density and richness, we fitted a third- and second-degree, respectively, polynomial mixed model using the log_10_ transformed density or ASV richness as the response variable and day, treatment, amount of *F. columnare* Fc7 added and the interactions between these as the explanatory variables. Sampling day was added as a random intercept term for each sampling unit. Statistical significance was evaluated by performing a Dunnett test on the estimated marginal means ratio difference between control and either phage- or antibiotic treatment at each sampling day, amount of *F. columnare* Fc7 added and treatment comparison.

The β-diversity was evaluated using the Bray–Curtis and Sørensen similarity. The similarity matrixes were obtained by taking the average of 100 similarity matrixes generated by random subsampling of the ASV-table to 26,448 reads^43,44^. The average Bray–Curtis and average Sørensen similarity matrixes were ordinated using principal coordinate analysis (PcoA). Statistical significance was evaluated with the mean of 100 permutational analysis of variances (PERMANOVA, 999 permutations).

Differential abundance analysis was performed on communities sampled at day 7 using corncob^45^, DESeq2^46^ and ANCOMBC^47^ using the absolute abundance. As input to all tree methods, we filtered out ASVs from the full dataset with a prevalence and total absolute abundance below 5% and 2500 ASVs/mL, respectively. Because different tools can identify different taxa as significant^48^, we conservatively defined ASVs identified by all three methods as having significantly different absolute abundances between the treatments.

Community assembly was investigated by quantifying the change in the similarity between communities of replicate microcosms per day (i.e. replicate similarity rate)^40^. The replicate similarity rate was determined as follows. First, the Bray–Curtis and Sørensen similarity was quantified for each pair of replicate microcosms on each sampling day. Next, we performed mixed linear regressions with similarity as the response variable and DPI, treatment type, amount of *F. columnare* Fc7 added and the interaction between these as the explanatory variables. Repeated sampling from the same unit was accounted for in the random effects term. The temporal slope rate was interpreted as a replicate similarity rate of change. Positive rates indicate that the community composition between two replicates became more similar over time, indicative of selection dominating community assembly. Negative rates, on the other hand, reflect that the replicates became less similar over time, indicating that drift is dominating community assembly. This similarity rate of change was estimated for each experimental group.

## Results

In this study, *Flavobacterium columnare* strain Fc7 was added at three different amounts (no, low or high) to microcosms containing a lake water bacterial community. The microcosms subsequently received either a bacteriophage- or antibiotic treatment or were left untreated. Throughout the result section, treatment refers to the treatment application. Each experimental condition was replicated five times, and the bacterial community was studied over one week.

**Figure 2:**
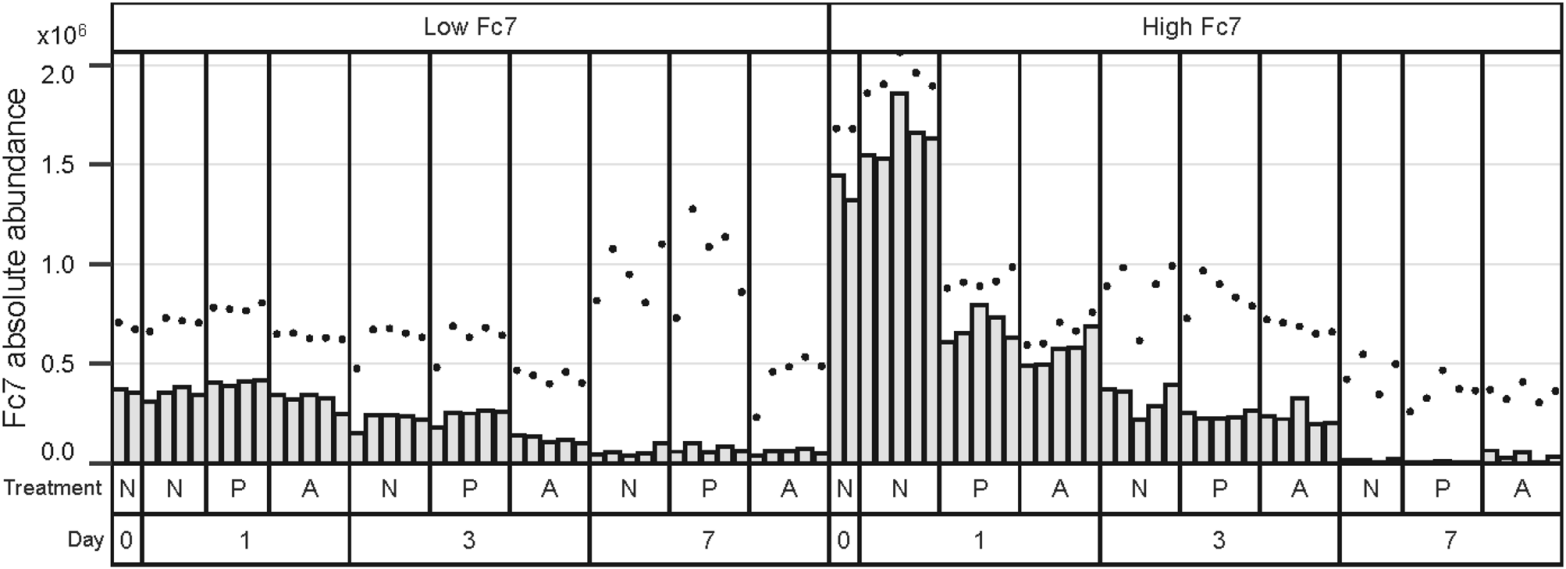
The absolute abundance of *Flavobacterium columnare* strain Fc7 (ASV 1) (10^6^ 16S rRNA gene copies/mL) in the microcosms added low and high amounts of *F. columnare* Fc7 at each sampling day. Points indicate the total bacterial density in each community (10^6^ cells/mL). Abbreviations; Treatment: N = no treatment, P = Phage treatment (FCL-2), A = Antibiotic (Penicillin).

The *F. columnare* Fc7 abundance decreased in all microcosms regardless of treatment type ASV1 was identified as the added *F. columnare* Fc7 population, and we scaled the relative ASV abundance with the bacterial density to obtain absolute ASV1 abundances. ASV1 made up, on average, 52.7 ± 0.1% and 82.2 ± 4.7% of the communities immediately after adding low and high amounts of *F. columnare* Fc7, respectively. Throughout the experiment, the *F. columnare* Fc7 abundance decreased in all microcosms regardless of the treatment, even in the control microcosms (Fig. 2). However, this observation was not due to the ineffective killing of *F. columnare* Fc7, as the decrease was more pronounced in the phage- and antibiotic-treated microcosms. For example, at day 1 in the microcosms added high amounts of *F. columnare* Fc7, we observed a 60% decline in *F. columnare* Fc7 absolute abundance in the phage- and antibiotic-treated bacterial communities compared to the control (Pairwise Wilcox test, p = 0.012). Furthermore, the effectiveness of the treatments was confirmed with live-dead staining, which showed that the living population was strongly reduced when phage- or antibiotic treatment was applied (living population at in microcosms added high amounts of *F. columnare* Fc7 at day 1; Control 75%, Phage 46%, Antibiotics 61%, see Supplementary Fig. 5). These declines indicate that both the phage- and antibiotic treatment effectively inactivated *F. columnare* Fc7 and that the strain was an unsuccessful invader.

### Bacterial density was impacted by the treatments

**Figure 3:**
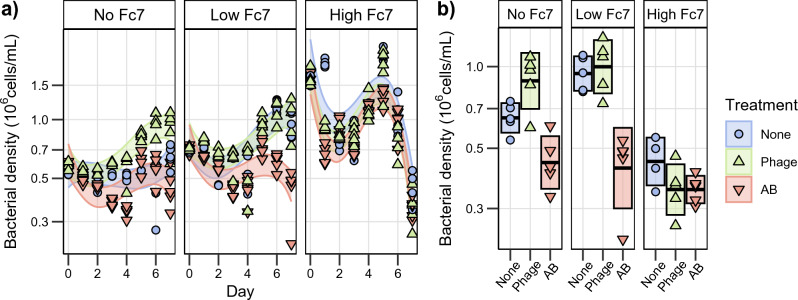
Changes in total bacterial density over time (y-axis is log_10_ scaled). (**a**) The bacterial community density (10^6^ cells/mL) in each microcosm over time (days). The shaded area indicates the 95% confidence interval of the model prediction. (**b**) The bacterial density at day 7. The box indicates the mean ± standard deviation. Colours and shapes indicate the treatment type. Treatment: None = no treatment, Phage = Phage treatment (FCL-2), AB = Antibiotic (Penicillin), No Fc7 = uninvaded, Low or High Fc7 = 24% or 190% increase in density after addition of *F. columnare* Fc7.

To compare how the phage- and antibiotic treatment affected the bacterial density, we fitted a mixed effect model with the bacterial density as the response variable and day, treatment (phage, antibiotic, none), amount *F. columnare* Fc7 added (no, low, high) and the interactions between these as explanatory variables. Sampling day per microcosm was defined as a random variable (Fig. 3a, R^2^ = 0.78, Supplementary Table 1). Statistical significance was determined by comparing the marginal mean estimates of the phage- or antibiotic treatment to the control at each day (Supplementary Table 2).

The effect of the phage treatment on the bacterial density varied depending on the amount of *F. columnare* Fc7 added. In the microcosms without *F. columnare* Fc7 added, the ratio in marginal mean bacterial density estimate between the phage treated and the control increased from 1.13 ± 0.15 (ratio ± SE) at day 0 to 1.50 ± 0.19 at day 7 (p < 0.001, Fig. 3b). When low amounts of *F. columnare* Fc7 were added to the microcosms, the phage treatment had no observable effect on the cell density, as the phage treated and the control microcosms had similar cell densities over time (p > 0.05 at all time points). However, in the microcosms added high amounts of *F. columnare* Fc7, the bacterial density ratio between the phage treated and control microcosms decreased from 0.78 ± 0.10 at day 0 to 0.72 ± 0.10 at day 7 (p = 0.03). In fact, all microcosms added high amounts of *F. columnare* Fc7 had a substantial decline in bacterial density from day 5 to 7, resembling a feast-famine response. We speculate that the density decline was not a result of the phage treatment but instead induced by a significant release of DOM due to the death of *F. columnare* Fc7 (see 'Discussion' section).

The antibiotic treatment negatively impacted the bacterial density. At day 7, the bacterial density was lower in the antibiotic-treated communities compared to the control, with a ratio of 0.76 ± 0.10 (p = 0.07) in the microcosms without *F. columnare* Fc7 added, 0.44 ± 0.06 (p < 0.001) in the microcosms with low amounts of *F. columnare* Fc7 added and 0.76 ± 0.10 (p = 0.09) in the microcosms with high amounts of *F. columnare* Fc7 added (Fig. 3b). Thus, compared to the detrimental effect of antibiotics, the impact of phage treatment on bacterial density was minor.

### Phage treatment had a negligible impact on α-diversity, whereas antibiotics drastically reduced it

**Figure 4:**
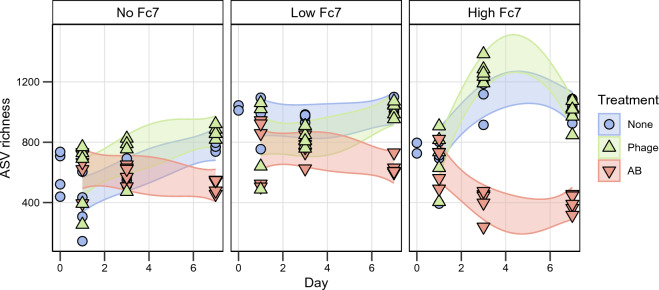
The ASV richness in each sample over time (days). Points are the observed richness, and the shaded area indicates the 95% confidence interval of the model prediction. Colours and shapes indicate the treatment type. Treatment: None = no treatment, Phage = Phage treatment (FCL-2), AB = Antibiotic (Penicillin), No Fc7 = uninvaded, Low or High Fc7 = 24% or 190% increase in density after addition of *F. columnare* Fc7.

To evaluate the treatment effect on the α-diversity, we determined the ASV richness (Fig. 4), Hill diversity of the first and second order, and evenness (Supplementary Fig. 6). To estimate the differences between the treatments and control, we fitted a mixed effect model with ASV richness as the response variable and DPI, treatment, amount of *F. columnare* Fc7 added, and their interaction as the explanatory variables. Sampling day was included as a random effect term (R^2^ = 0.80, Supplementary Table 3). Post-hoc comparisons are summarised in Supplementary Table 4.

We found no statistical evidence that phage treatment decreased richness. The exception was at day 3, where the richness was on average 1.12 × higher in the microcosms added high amounts of *F. columnare* Fc7 receiving phage treatment than in the controls (Supplementary Table 4). The antibiotic treatment decreased richness. This reduction was particularly evident at day 7, where the richness was on average 0.65x, 0.62x, and 0.38 × lower in the antibiotic-treated than in control microcosms in the microcosms without *F. columnare* Fc7 (p-value < 0.01), added low amounts of *F. columnare* Fc7 (p-value < 0.001) and added high amounts of *F. columnare* Fc7 (p-value < 0.001), respectively.

The observation that phage treatment had negligible effects, while antibiotics reduced the ASV richness, was also found for Hill diversity of order 1 and 2 and evenness (Supplementary Fig. 6). In conclusion, the antibiotic treatment caused a loss of biodiversity. Notably, the phage treatment did not decrease α-diversity, indicating that the bacterial populations were resilient to this disturbance.

### Bacterial community composition was similar between the phage and control treatment

**Figure 5:**
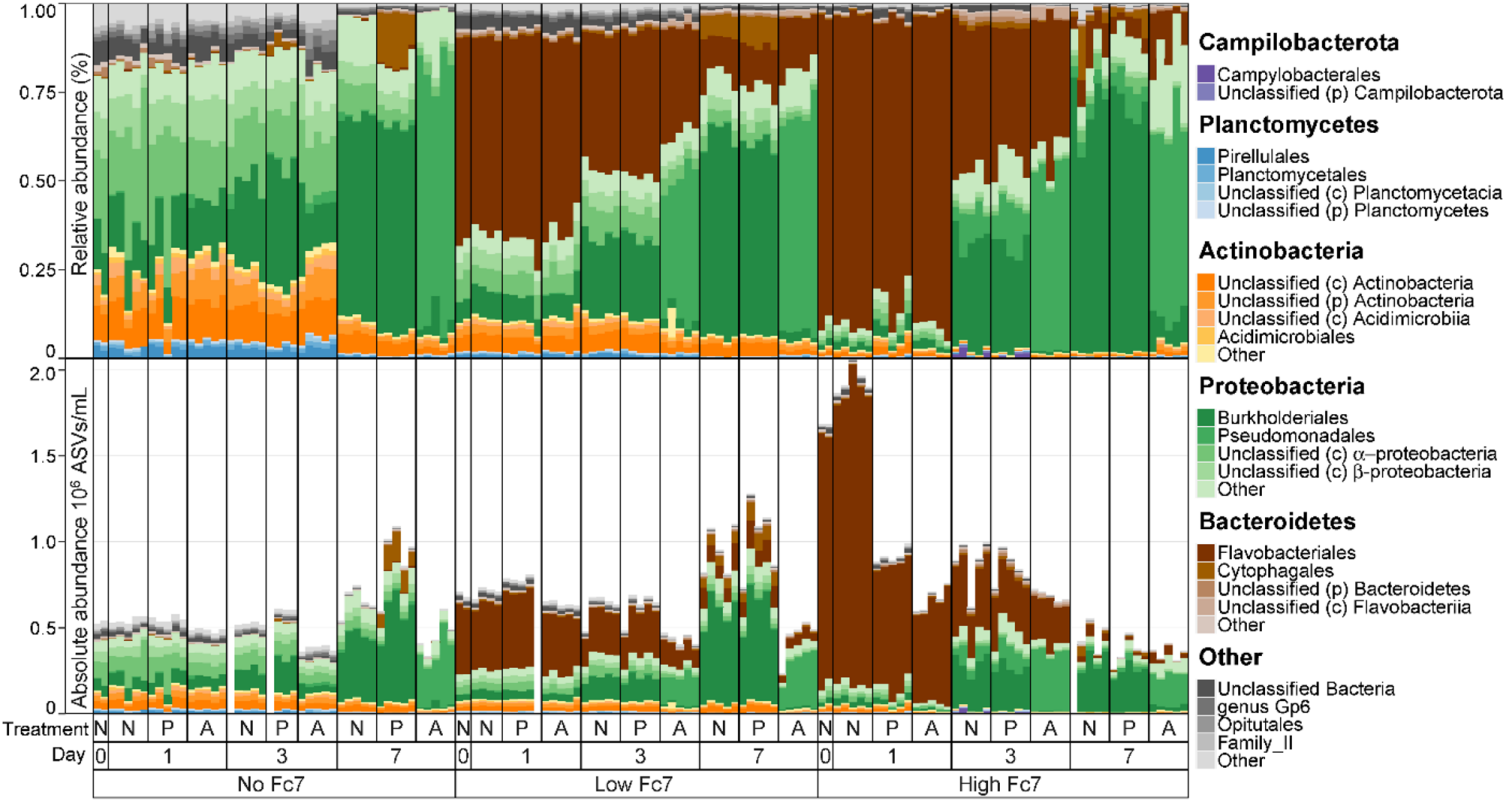
The bacterial community composition at the order level grouped according to bacterial phyla. Only the four most abundant orders are shown, and the rest are grouped as other. Community composition was evaluated as the relative- (upper panel, %) and absolute abundance (bottom panel, 10^6^ ASV copies/mL). Treatment: N = no treatment, P = Phage treatment (FCL-2), A = Antibiotic (Penicillin), No Fc7 = uninvaded, Low or High Fc7 = 24% or 190% increase in density after addition of *F. columnare* Fc7.

**Figure 6:**
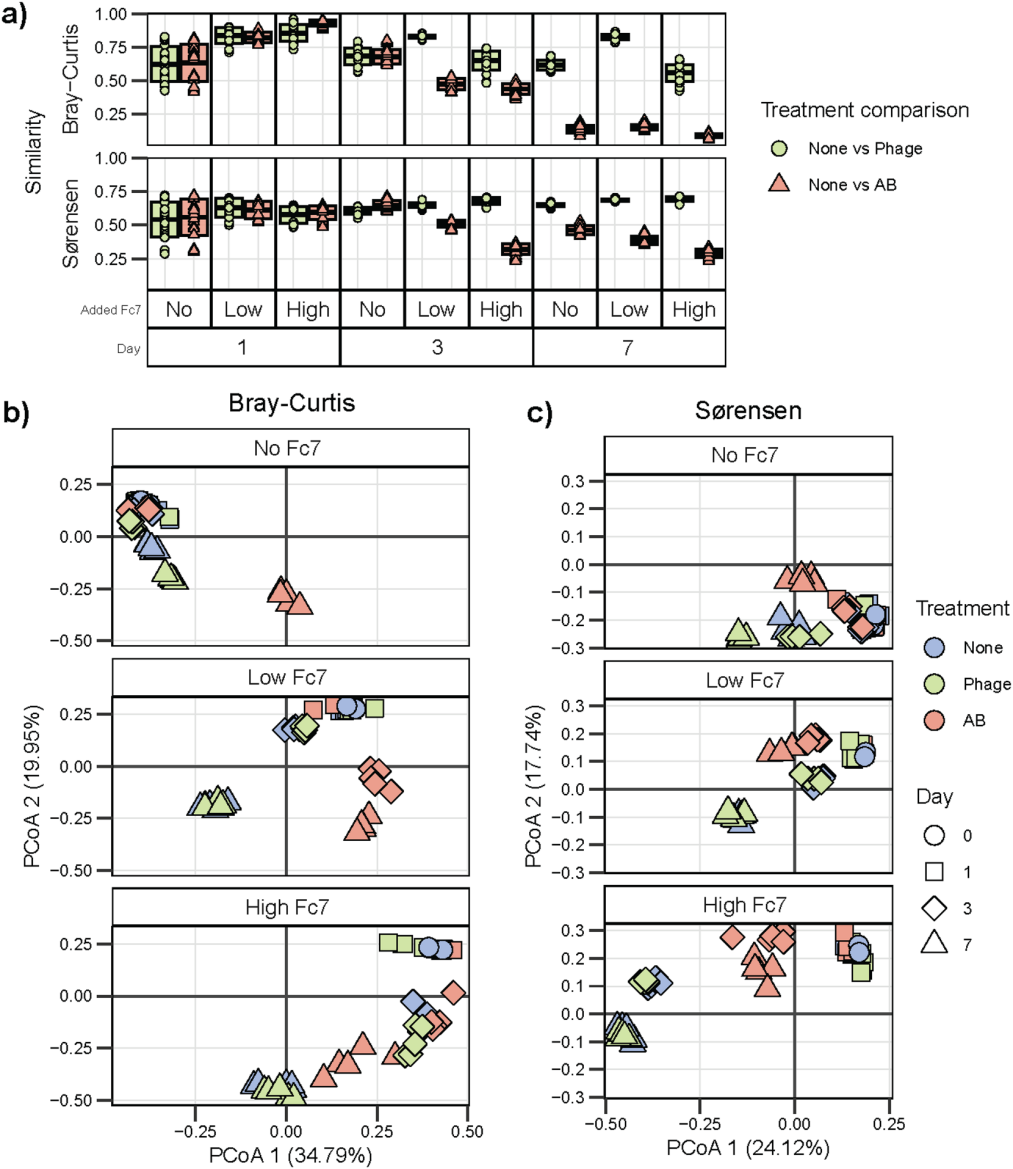
Bray–Curtis and Sørensen similarity between samples indicated that communities changed over time. (**A**) Bray–Curtis (upper) and Sørensen similarity (lower panel) comparing the control (none) and the treatments phage or antibiotic, at each sampling day and level of *F. columnare Fc7* added. Colour and shape indicate comparison. (**B**,**C**) Bray–Curtis and Sørensen based PcoA ordinations of samples taken at day 0, 1, 3 and 7. Each plot is a single ordination but is separated based on the amount of *F. columnare* Fc7 added for clarity. Colours and shapes indicate the treatment type and sampling day. Treatment: None = no treatment, Phage = Phage treatment (FCL-2), AB = Antibiotic (Penicillin), No Fc7 = uninvaded, Low or High Fc7 = 24% or 190% increase in density after addition of *F. columnare* Fc7.

The bacterial community composition was similar between the phage treatment and the control when evaluating composition at the order level (Fig. 5) and by PCoA ordinations based on both Bray–Curtis and Sørensen similarity (Fig. 6). After one day, there was no significant difference in the community composition between the phage treatment and untreated control, regardless of the amount of *F. columnare* Fc7 added (Bray–Curtis and Sørensen based PERMANOVA p > 0.05, Supplementary Table 5). Thus, the data is suitable for studying the effects of the treatments on bacterial community succession.

The phage treatment did not impact the community succession in microcosms where *F. columnare* Fc7 had been added, as there were no significant differences between the phage-treated and control communities at day 7 (PERMANOVA p > 0.05). However, for the microcosms without *F. columnare* Fc7, there was a significant difference in the community composition based on both ASV abundance (Bray–Curtis PERMANOVA r^2^ = 0.77, p = 0.009) and presence-absence (Sørensen PERMANOVA r^2^ = 0.26, p = 0.009).

Differential abundance analysis conducted on samples from day 7 identified 18 ASVs (8 genera) with a ratio of absolute abundance between the phage treatment and control below 0.2 or over 5 (Supplementary Fig. 7). Of these, 14 were identified in the microcosms without *F. columnare* Fc7, six in microcosms added low amounts of *F. columnare* Fc7, and only two in the microcosms added high amounts of *F. columnare* Fc7. These differences indicate that the phage treatment impacted the uninvaded microcosms the most. Of particular interest was an ASV belonging to the genus *Aquirufa,* with an absolute abundance 227 times higher in the phage treated (1.2 × 10^5^ ± 4.0 × 10^4^ASVs/mL) than the control (530 ± 459ASVs/mL) microcosms without *F. columnare* Fc7 added. Despite significant differences, the average Bray–Curtis similarity between the phage-treated and control communities only changed slightly from 0.77 ± 0.14 at day 1 to 0.67 ± 0.12 at day 7 (Fig. 6a). Thus, we conclude that the phage treatment had no impact on community composition and succession when the phages bacterial host was added, but that minor changes were induced when the phage host was absent.

### Antibiotic treatment caused a significant disturbance event in the community

In contrast to the phage treatment, antibiotics caused the community composition to change significantly compared to the control microcosms (Fig. 6). At day 7, the community composition significantly differed between the antibiotic-treated and control microcosms (PERMANOVA, r^2^ range 0.52–0.84, p < 0.05 for both Bray–Curtis and Sørensen). These changes are evident in the average similarity between communities from the antibiotic-treated and control microcosms. There was a 6 × reduction in Bray–Curtis similarity (0.79 ± 0.15 at day 1, 0.13 ± 0.035 at day 7) and a 1.5 × reduction in Sørensen similarity (0.59 ± 0.095 at day 1, 0.38 ± 0.078 at day 7) (Fig. 6a).

Differential abundance analysis identified 122 ASVs (29 genera) with a ratio in absolute abundance between the antibiotic-treated and control communities below 0.2 or over 5 (Supplementary Fig. 7). Thus, there were 4.2 × more ASVs with such a substantial difference in the antibiotic-treated than the phage-treated microcosms. Interestingly, of the 122 ASVs, all ASVs classified as *β-proteobacteria* (68 ASVs) had higher absolute abundances in the control microcosms, while all classified as *γ-proteobacteria* (11 ASVs) had higher absolute abundances in the antibiotic-treated microcosms. These 11 ASVs belonged to the genus *Pseudomonas* which contains many pathogenic bacterial strains^49^.

In conclusion, the antibiotic treatment caused significant disturbances that the bacterial communities did not recover from after seven days and caused a bloom of *Pseudomonas*.

### Phage treatment did not affect the bacterial community assembly, while antibiotics caused a shift from selection to drift

We investigated the community assembly within each experimental condition by calculating the change in similarity between replicate microcosms over time (i.e. similarity rate) as described in Gundersen et al. 2021^40^. Increasing similarity rates indicate that the deterministic process selection dominates community assembly. In contrast, decreasing similarity rates indicate an increased contribution of the stochastic process ecological drift.

**Figure 7:**
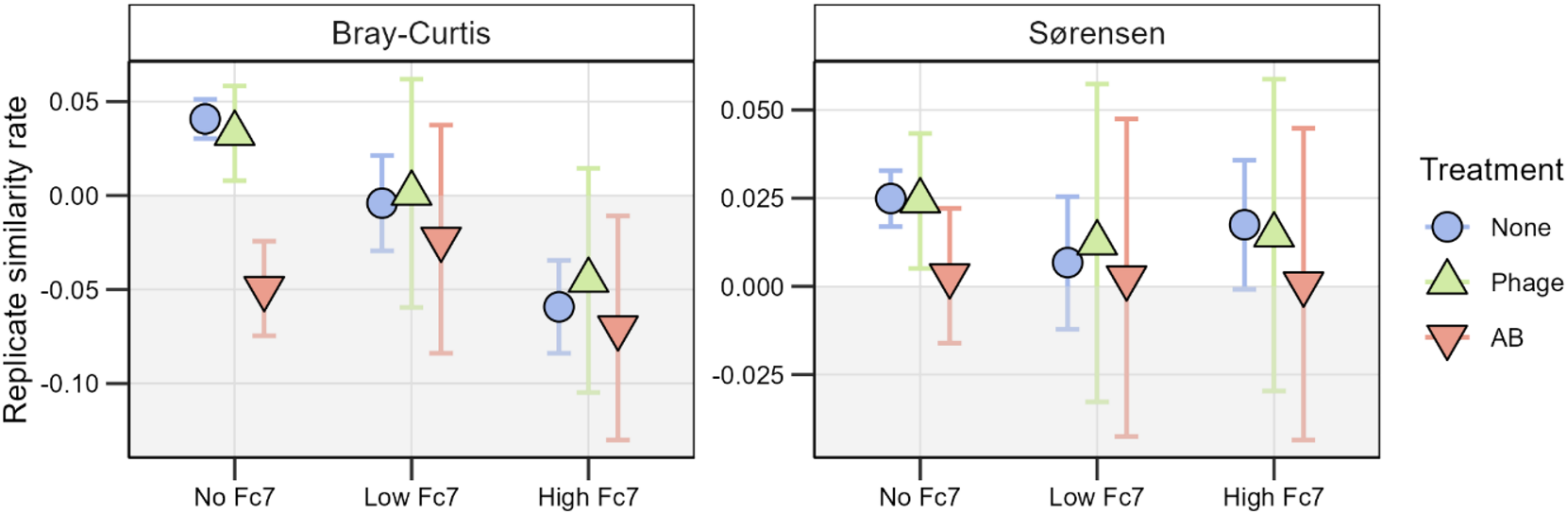
Estimated replicate similarity rate for experimental group based on (**a**) Bray–Curtis and (**b**) Sørensen similarity. The error bars are the 95% confidence interval of the estimated similarity rate of change. Colours and shapes indicate the treatment type. Treatment: None = no treatment, Phage = Phage treatment (FCL-2), AB = Antibiotic (Penicillin), No Fc7 = uninvaded, Low or High Fc7 = 24% or 190% increase in density after addition of *F. columnare* Fc7.

We used this assembly framework with both the Bray–Curtis and Sørensen similarity (Fig. 7, Supplementary Fig. 8). We first examined the microcosms without *F. columnare* Fc7 added to evaluate the effect of phage- and antibiotic treatment on community assembly when no phage host was present (i.e. not considering the effect of adding *F. columnare* Fc7). The phage treated and control microcosms had comparable positive similarity rates, with the average varying between 0.033 and 0.040/day for the Bray–Curtis and 0.024–0.025/day for the Sørensen similarity rate. These positive rates indicated that the communities in replicate microcosms became more similar over time and were thus primarily structured by selection. On the other hand, the antibiotic-treated microcosms had a negative similarity rate based on Bray–Curtis (− 0.049/day), while the Sørensen-based was slightly positive (0.003/day). Thus, when antibiotics were added, the community composition was structured by drift, with some selection at the ASV inventory level (Fig. 7).

Next, we determined if treatment and amount of *F. columnare* Fc7 added combined affected community assembly. At each level of added *F. columnare* Fc7, the Bray–Curtis and Sørensen similarity rates of the phage-treated microcosms were not significantly different from the control microcosms. However, the average similarity rate decreased with increasing amounts of *F. columnare* Fc7 added for both the phage-treated and the control microcosms. This effect of *F. columnare* Fc7 amounts added was not observed for the antibiotic-treated microcosms, where the Bray–Curtis similarity rate was clearly negative regardless of amounts of *F. columnare* Fc7 added (no = − 0.049, low = − 0.023, high = − 0.070). Thus, the treatment (phage and antibiotic) and amounts of *F. columnare* Fc7 did not have an additive effect.

## Discussion

Comprehending the ecological impacts of phage therapy is vital due to the increased interest in applying this technology^18,50^. A concern is a lack of understanding of how phages impact the resident bacterial community. From one perspective, it is desirable to use therapeutic agents that minimally impact the microbiome when treating bacterial infections in animals or humans to ensure stability in the bacterial community. Currently, this knowledge gap hinders clinical approvals for using phages in humans due to the potential disturbance it can cause to the microbiome^51^. Furthermore, phages are introduced into ecosystems through, for example, water released from aquaculture facilities^18^ and when sprayed over agricultural fields^50^. Thus, we must elucidate how the bacterial communities in such phage-receiving ecosystems respond to phage exposure. As such, the study of the impacts of phage therapy is not just a scientific pursuit but also relevant for society with the aim to ensure safety and sustainability of ecosystems.

Most studies investigating the effect of phage treatment on the properties of the resident community have been performed in situ (e.g. human and animal gut). Although more realistic, these ecosystems contain many unknown or uncontrollable variables that can mask changes in the resident bacterial community^52^. Our experiment aimed to reduce the environmental complexity and minimise within-group variability by bringing a planktonic lake community into controlled laboratory conditions. The observed community composition was highly similar between biological replicates at day 1, indicating that our goal of creating replicate communities was successful.

The aim of this study was to investigate how phage treatment affected community structure. We were particularly interested in understanding how the amount of the bacterial host of the phage affected the outcome. The release of resources, such as mineral nutrients and DOM, is proportional to the number of hosts lysed. We hypothesized that over a threshold, lysis products would induce a change in the structure of the resident bacterial community. We therefore introduced the phage host *F. columnare* Fc7 at two different levels.

As the added *F. columnare* Fc7 did not establish in the system, the invasion was unsuccessful. Thus, also the resident community in the control and treatment groups without addition of phage experienced an increase in available resources due to the death of *F. columnare* Fc7. To account for this unsuccessful invasion our analytical approach was to compare the treated and control microcosms with the same level of *F. columnare* Fc7 added. These comparisons reflect the effects of adding phage or antibiotic to increase the death rate of a declining population. Had the *F. columnare* Fc7 population successfully established in the control microcosms, the results may have been different.

Furthermore, in the current experiment, the *F. columnare* Fc7 invasion was performed only an hour before the treatments were applied. It is unrealistic that *F. columnare* Fc7 formed any meaningful interactions with the resident community in that timeframe. Consequently, we could not evaluate the possible changes phage treatment induces in the bacterial interaction networks. It has been demonstrated that phages can result in cascading effects in the interaction network of a 10-species synthetic community^53^. Therefore, future studies should investigate the impact of removing an established population from the resident community.

Our comprehensive study of the bacterial communities showed that phage treatment had a marginal biological effect on community properties such as density, α-diversity, composition, succession, and assembly, compared to the control. Most analyses showed no statistically significant changes between the phage-treated and control microcosms. When *F. columnare* Fc7 was added to the community, statistically significant effects of phage treatment were detected in only a few cases, indicating that the treatment had little effect on community structure. We observed that the bacterial density in the phage-treated microcosms was halved after 1 day compared to the control, indicating that the phage was successful in targeting and lysing the *F. columnare* Fc7 cells. Quantification of the free phage population would have provided a better understanding of the phage dynamics in this experimental setup. On day 3, ASV richness was 12% higher in the phage-treated microcosms compared to the control microcosms where high levels of *F. columnare* Fc7 were added. However, this was the only occasion when there was a significant difference in diversity. Therefore, when evaluating the totality of the analyses performed, it appears that phage treatment induces only minor to negligible effects on community characteristics when the phage host is present.

Furthermore, we found no evidence for a greater effect of phage treatment on community structure, when more hosts were added. In general, no significant differences were detected between the phage-treated and control microcosms. This applies for both low and high levels of *F. columnare* Fc7 added. This is probably related to the fact that *F. columnare* Fc7 also died in the control microcosms, and future studies should challenge these findings with a more successful invader.

In contrast to our hypothesis, we observed the largest impact of the phage treatment on the bacterial community characteristics when no phage host (i.e. *F. columnare* Fc7) was present. When the microcosms without *F. columnare* Fc7 were added phages the density was on average 38.4% higher than in the control, and we observed a significant change in the community succession that resulted in changes in ASV inventory and relative abundance compared to the control. The changes were mainly driven by a substantial increase in a single ASV classified as *Aquirufa*. This genus is part of the order *Cytophagales,* known to be efficient degraders of biopolymers such as proteins, DNA and RNA^54^. It is known that phages can function as a substrate for heterotrophic bacterial growth^34^. Noble et al. 1999 observed that bacterial density increased after adding a phage cocktail to a bacterial community. The authors concluded that the phage particles stimulated the growth of non-infected heterotrophic bacteria^55^. In a follow-up study, they radiolabelled viral components and subsequently found them incorporated into the bacterial biomass^56^. Phages are essential in the microbial loop by increasing DOM turnover through the lysis of bacteria (i.e. viral shunt). Our observations indicate that some non-target resident bacteria benefit from viral decay. Thus, exploring how viral decay contributes to the microbial loop would be fascinating and appears to be a knowledge gap.

We observed that the bacterial density fluctuated in a feast-famine response manner in the microcosms added high amounts of *F. columnare* Fc7, regardless of the treatment. The feast-famine response occurs when communities experience a surge in available resources, leading to an increase in density, followed by a decline due to famine after resource consumption^57^. The *F. columnare* Fc7 population declined drastically during the first days. When bacteria lyse, DOM is released, which can be consumed by the resident community^58,59^. From day 3 to 5, we observed a doubling in cell density, which might be explained by a feast on DOM released from lysed *F. columnare* Fc7 cells. Following the depletion of DOM, we observed a substantial 4.5-fold decrease in density. This decrease likely occurred as the carrying capacity of the system could not sustain the peak in population density, which led to famine-induced mortality. We speculate that this famine response was stronger in the phage treatment, possibly due to the initial pulse in resources at day 1 due to lysis of *F. columnare* Fc7. This stronger response may explain why we observed a 21% lower bacterial density on day 7 in the microcosms added high amounts of *F. columnare* Fc7 and phage treatment as compared to the control. Our understanding of such feast-famine responses in controlled experimental settings is still poor and further investigation of these dynamics would be beneficial to the field.

Increased amounts of *F. columnare* Fc7 added was accompanied by a shift from selection to ecological drift dominating the community assembly. Zhou et al. 2014 hypothesised that nutrient disturbances should enhance stochastic community assembly due to reduced niche selection and growth of the rare biosphere^60^. They found evidence for their hypothesis using vegetable oil as a nutrient spike. Through 16S rRNA gene sequencing and flow cytometry, we showed that the relative and absolute abundance of *F. columnare* Fc7 declined, possibly leading to a substantial increase in DOM. Thus, we support their hypothesis by showing that dead bacterial cells increase the contribution of stochastic processes. Increased stochasticity results in more unpredictable changes at the community level and, consequently, replicate microcosms diverge from each other. Hence, researchers should carefully consider how much of the invader they will add when planning invasion studies. Our observations indicate that too high invader concentrations can result in a feast-famine response and increased ecological drift.

The antibiotic treatment functioned well as a positive disturbance control. The antibiotic treatment caused a substantial decrease in bacterial density, reduced α-diversity, significantly changed community composition and enhanced stochastic community assembly. These characteristics are indicative that the antibiotic treatment caused a severe disturbance. We conclude that the resident community had little resistance to this disturbance, as it was strong enough to push the community out of its stable state^61,62^.

The bacterial density declined significantly in the antibiotic-treated communities compared to the control. This decline and a loss in ASV richness indicate that several bacterial populations died. This bacterial death has two effects: increased DOM release and reduced niche competition. Both effects are characteristic for conditions that select for opportunistic r-strategic bacteria that respond to high resource availability by rapidly increasing their growth rate leading to a numeric response in cell density^59^. Most pathogenic bacteria are classified as r-strategic bacteria^59^. Intriguingly, the antibiotic treatment significantly increased ASVs classified as *Pseudomonas* (*γ-proteobacteria*). This genus is associated with r-strategic organisms^63^ and contains many pathogenic bacteria, such as *P. aeruginosa* and *P. fluorescens*^49^. Thus, our observations show that the antibiotic treatment created an environment that allowed opportunistic bacteria to bloom. This discovery is concerning due to the possibility that antibiotic treatment may result in dysbiosis and selection for antibiotic-resistant pathogenic bacteria.

## Conclusions

Our study investigated the impact of phage treatment on resident community structure. This study is the first to explore how the density of the phage host impacts the effects of phage treatment on community structure. The amount of host (*F. columnare* Fc7) had an impact. We found that a single FCL-2 phage treatment had a negligible impact on community diversity and composition when the host *F. columnare* Fc7 was added to the community. Interestingly, we observed significant effects of phage addition when *F. columnare* Fc7 was absent, mostly driven by an increase in the abundance of *Aquirufa sp*. Further investigations should explore the underlying mechanisms for this observation. Nevertheless, when changes due to the phage treatment were observed, they were minor compared to the detrimental impacts of the antibiotic treatment. Antibiotics resulted in a substantial decline in bacterial density and α-diversity, altered the community composition and triggered a bloom of opportunistic bacteria. Such drastic changes were not observed for phage treatment in the presence of the bacterial host. These findings are relevant for treatment of humans and for industries such as aquaculture, agriculture, and wastewater management, as they benefit from stable and functional microbial communities. As the phage treatment induced only minor changes to community structure, our observations indicate that phage therapy is a safer and superior alternative to antibiotics for therapeutic use in host microbiomes, such as the human gut.

### Supplementary Information

Supplementary Information.

## Data Availability

All data generated from flow cytometry (FSC files) are available at https://figshare.com/projects/Flow_cytometry_data/152886. The Illumina sequencing reads are deposited at the European Nucleotide Archive (accession number PRJEB59722).
