# Supplementary material for: The impact of phage treatment on bacterial community structure is minor compared to antibiotics
Source: Sci Rep. 2023 Nov 29;13:21032. doi: 10.1038/s41598-023-48434-5 (PMC10687242; doi:10.1038/s41598-023-48434-5)
Supplement: Supplementary file 1 — Supplementary Information. [file 41598_2023_48434_MOESM1_ESM.pdf]

Supplementary materials, methods and results for: *The impact of phage treatment on bacterial community structure is minor compared to antibiotics*

Authors:

Madeleine S. Gundersen, Alexander Willi Fiedler, Ingrid Bakke, Olav Vadstein

Corresponding author: Olav Vadstein (olav.vadstein@ntnu.no) & Madeleine S Gundersen (madeleine.gundersen@ntnu.no)

Supplementary methods

Phage enrichment and concentration estimates

The phage FCL-2 targeting *Flavobacterium columnare* Fc7 was used for the phage treatment [1].

FCL-2 selectivity and virulence towards *Flavobacterium columnare* strain Fc7

Upon receiving the phage, we confirmed its virulence towards the *F. columnare* strain Fc7 using the soft-agar overlay technique and spot testing [2]. For that, 1mL of *F. columnare* Fc7 culture in the exponential phase was mixed with 3mL 0.75% TYES agar (50°C) and plated on a 1.5% TYES agar plate. After 1h of incubation at room temperature, 5µL droplets of the phage stock were added to the plate and incubated at room temperature for one day. The formation of plaques in the bacterial lawn indicated that *F. columnare* strain Fc7 was susceptible towards phage FCL-2 (Supplementary Figure 1).

a) b)

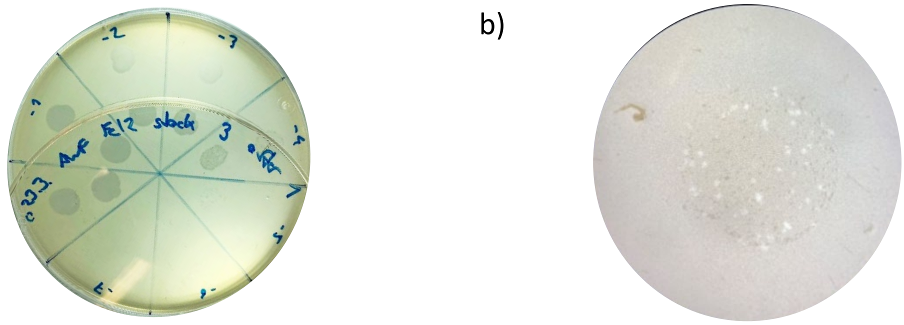

Supplementary Figure 1: a) Phage FCL-2 stock concentration spot test with Fc7. b) FCL-2 lysed the F. columnare Fc7 cells leaving clear spots on a soft-agar overlay agar plate. Magnified x25

FCL-2 phage stock preparation

After confirming virulence towards *F. columnare* Fc7, phage FCL-2 was enriched using the soft-agar overlay method by mixing 1mL of an *F. columnare* Fc7 culture in the exponential phase with 100µL of phage-stock and 3mL 0.75% TYES agar and pouring it on a TYES agar plate (1.5% agar). Plates were incubated overnight at room temperature. The phages were isolated by scraping off the top-agar layer and suspending the layer in sterile SM-buffer (5.8 g/L NaCl, 50 mL/L Tris buffer (1 M, pH 7.5), 2 g/L $MgSO_4$ * 7 H_20) at a ratio of approximately 1:1 (v/v) top agar and SM-buffer. After vortexing the suspension, it was centrifuged for 10 minutes at 10 000xg at room temperature, and the supernatant was filtered through a 0.2µm filter. Next, the phage

enrichment was pelleted by centrifugation at 22 000xg at 4°C for at least 8h. Finally, the supernatant was removed, and the pellet was resuspended in 10mL of SM-buffer before it was filtered through a 0.2µm filter to ensure bacterial sterility.

The concentration of phage FCL-2 was determined by serially diluting the phage stock with SM-buffer and adding 5µL of the dilutions to soft-agar plates. Plaques were counted after overnight incubation at room temperature to determine the phage concentration. The phage stock was determined to contain 10^{10} PFU/mL.

16S rRNA gene amplicon sequencing quality and normalisation

The amplicon library contained 4849 ASVs and 150 samples (143 water samples + 7 controls). Inspection of the control samples indicated that 127 ASVs were contaminants, and these were removed from the dataset. Additionally, we removed 4 ASVs identified as archaeal and another 127 identified as *Chloroplast*. Two samples (DNA_66.C.P.2.D3_S17, DNA_29.T.N.5.D1_S5) were of low quality due to low sequencing depth and were removed from the dataset. After the removals, the high-quality dataset contained 7 894 016 sequencing reads and 4630 ASV. The 141 samples had an average sequencing depth of 55 985±17 363 (mean±SD) reads. Rarefaction curves indicated that most samples had flattened off at the minimum sampling depth of 26 448 reads, with an average slope of 0.0084±0.004 (**Supplementary Figure 2**).

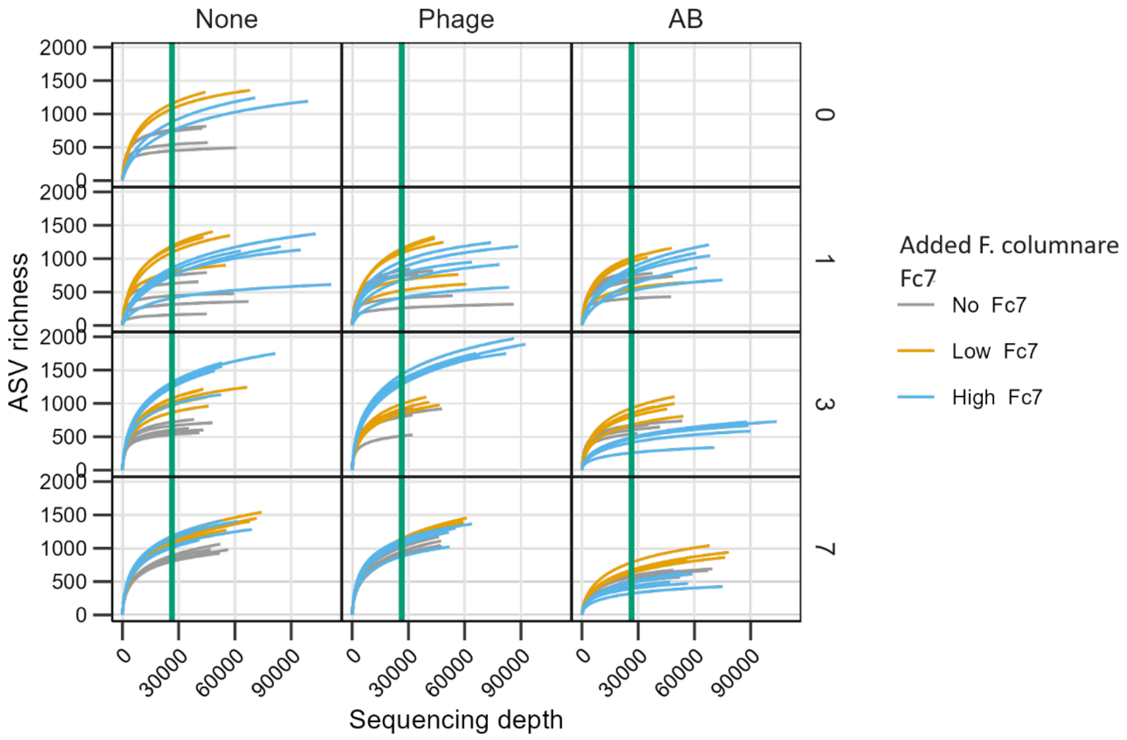

Supplementary Figure 2: *Rarefaction curves for all samples coloured according to the amount of F. columnare Fc7 added. The samples are organised based on the treatment type and sampling day. The solid vertical line indicates the sequencing depth chosen for normalisation at 26 448 reads.*

For α-diversity analysis, the dataset was normalised by scaling each sample to 26 448 reads before each sample was rarefied to 26 447 reads to ensure equal sampling depth. 69 ASVs were removed from the dataset due to normalisation. Before analysis, we inspected the normalised

dataset to ensure it reflected the full dataset satisfactorily. First, we inspected the correlation between the normalised and full dataset regarding Hill diversity numbers (N_{order}) of order 0 and 1 [3] (**Supplementary Figure 3**a). The correlation coefficient was 1 for N_1 and 0.92 for N_0. While the linear regression slope between the normalised and full dataset was 0.98 for N_1, it was only 0.67 for N_0 with increasing deviation from the 1:1 ratio with higher sample ASV richness (N_0). These highly rich samples had a higher sequencing depth (data not shown). Thus, while only 69 ASVs were lost, highly diverse and deeply sequenced samples lost more of their rare taxa due to the normalisation method.

Next, we visually inspected the community composition differences through PCoA plots based on the Bray-Curtis and Sørensen similarity (**Supplementary Figure 3**b and c). For the Bray-Curtis-based ordinations, the sample-clustering was highly similar between the normalised and full-dataset, while it was more condensed for the Sørensen similarity. Thus, the normalised dataset did indeed represent the core-microbiome stronger. However, the datasets were deemed to reflect the overall trends satisfactorily.

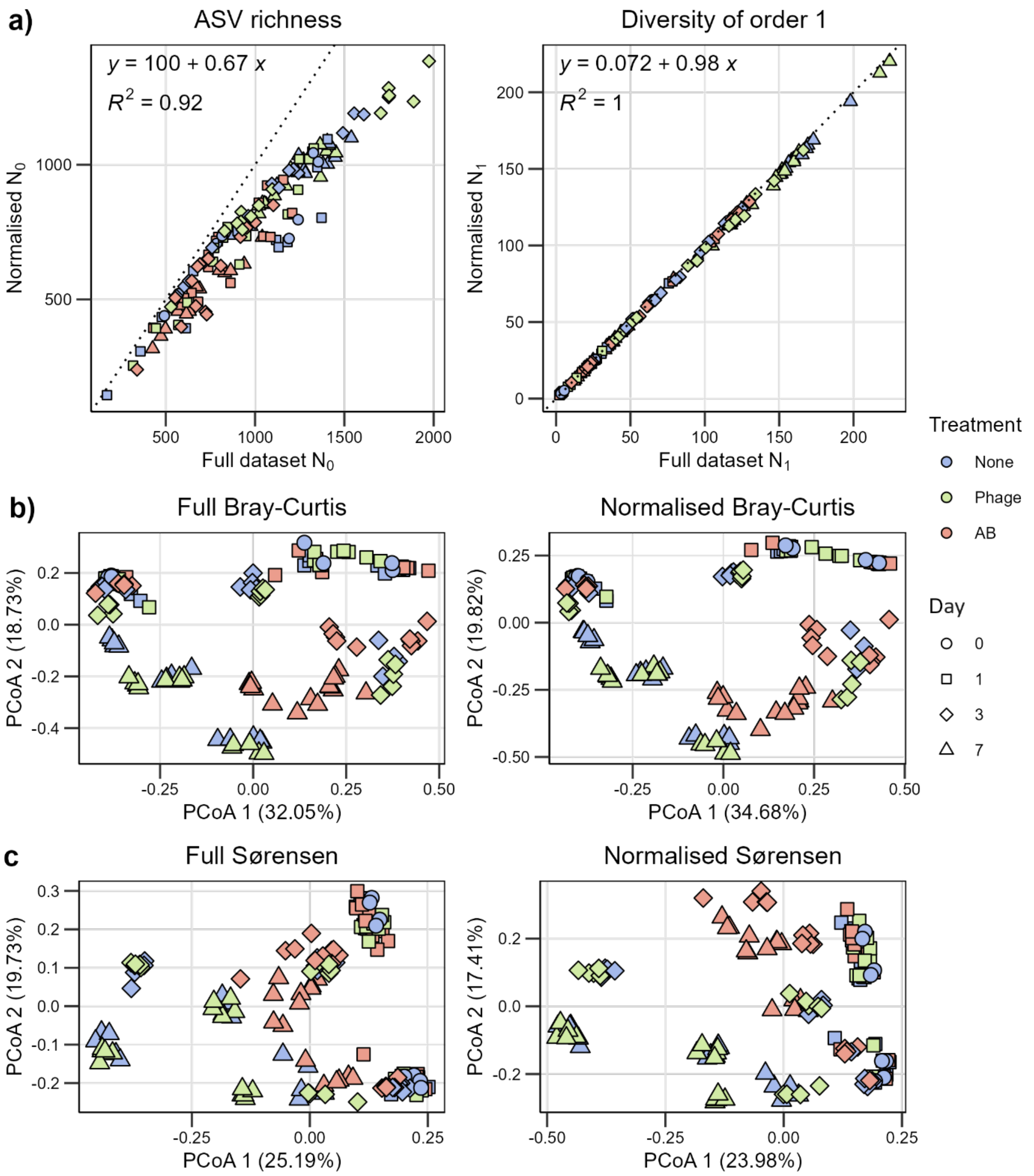

Supplementary Figure 3: *Comparisons of the normalised and full dataset in terms of a) Hill diversity of order 0 and 1 (N_0=richness, N_1=exponential Shannon), PCoA ordination based on b) Bray-Curtis similarity and c) Sørensen similarity. The normalised dataset contained 4561 ASVs and had a total loss of 69 ASVs compared to the full dataset. Colours indicate the treatment type and shape the sampling day. Abbreviations; Treatment: None = no treatment, Phage = phage treatment (FCL-2), AB = antibiotics (Penicillin).*

Quantification of bacterial density using flow cytometry

The total bacterial density and the proportion of living- dying, and dead cells were estimated throughout the experiment. The total bacterial density was quantified from fixated samples after the experiment was conducted, while the live-dead estimations were conducted daily from fresh samples. For both types of acquisitions, samples were diluted in 0.2μm filtered phosphate-buffered saline (PBS, 1x) to obtain stable sample aquation and dilute background noise present in the sample.

Total bacterial density

Each sample was first diluted to reduce occurrences of coincidence events. Next, SYBR green II stock (10 000x) was diluted in 0.2μm filtered PBS (1x) to 200x. This solution was used to stain samples to a final concentration of 2x. Stained samples were incubated for 15 minutes in the dark at 37°C before analysis. Five samples were stained at a time and data were acquired before staining more samples to ensure a stable fluorescent signal.

Data were collected using the blue laser (488 nm) with detection in BL1 (530±30 nm) and BL3 (>630 nm) using a BL1 threshold of 400-800 (depending on the sample). Instrument voltages were as follows; FSC 320V, SSC 340V, BL1 420 V and BL3 400V and YL2 620V. Data was acquired by running 110μL of the sample at a 100 μL/min flow rate. Each sample was vortexed before data acquisition. 0.2μm filtered PBS and 0.2μm filtered lake water were used as negative controls, and the Fc7 as a positive control.

All samples were gated similarly with minor modifications to each sample gate to ensure high-quality data (**Supplementary Figure 4**). The gating strategy was as follows: First, a stable signal collection was identified in the time-histogram plot, and the stable events were gated. Then all co-coincidence events were excluded by removing events with a higher BL1-A (area) than BL1-H (height) signal. Lastly, background noise was identified by comparing 0.2μm filtered lake water with non-filtered lake water and was gated out. The remaining events were identified as the bacterial community.

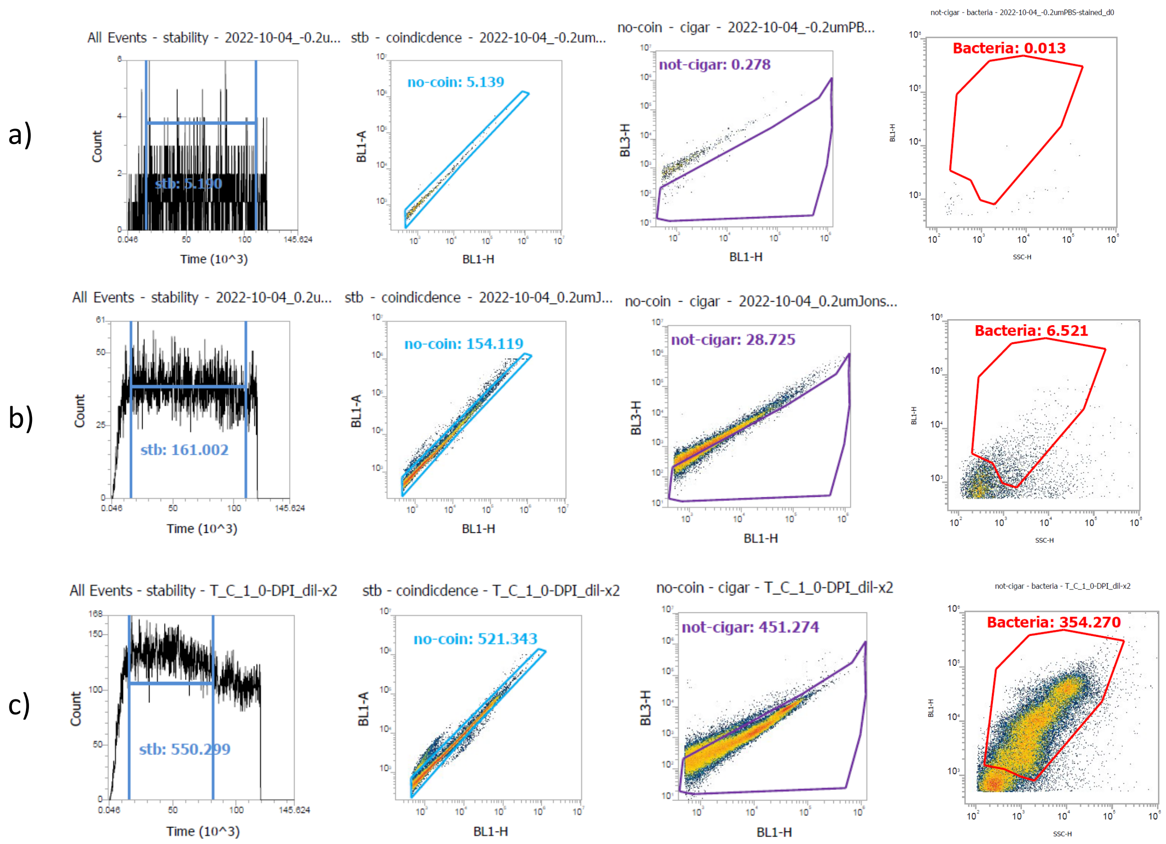

Supplementary Figure 4: *Gating strategy for total bacterial density quantification. A stable signal was first selected in the "stb" gate. Then all coincidence events were removed by only keeping events in the "no-coin" gate. We kept events in the "not-cigar" gate based on the negative controls and then gated out the bacterial populations in the SSC-H vs BL1-H scatterplot. a) 0.2µm filtered PBS solution was used as a negative control and for diluting the bacterial communities. b) 0.2µm filtered lake water and c) The bacterial community in the microcosms (here showing reactor replicate one from the control group added low amounts of F. columnare Fc7 (T-C-1) at 0 DPI).*

Living-, dying- and dead density quantification

For quantifying the living-, dying- and dead- bacterial population, we stained 1mL of the sample within one hour of sampling daily from replicate 3 in each experimental group. SYBR green I (10 000x, Invitrogen) and propidium iodide (PI, 20 mM, Invitrogen) were diluted in 0.2µm filtered PBS (1x) to a final concentration of 100x and 0.4mM. Each sample was stained with this solution giving a final dye concentration of SYBR green I (1x) and PI (0.4µM). SYBR green I and PI bind to DNA and emit a green and red fluorescent signal if exited at 488 nm, respectively. SYBR green I

can enter all cells, while PI only enters membrane-compromised cells (i.e. dying- or dead cells). Samples were incubated for 15 minutes in the dark at 37°C with the stain before acquiring data.

Data were collected using the blue laser (488 nm) with detection in BL1 (530±30 nm), BL3 (>630 nm) and YL-2 (620±15 nm) using a BL1 threshold of 400-800 (depending on the sample). Instrument voltages were as follows; FSC 320V, SSC 340V, BL1 420 V and BL3 400V and YL2 620V. Samples were acquired by running 110μL sample at a 100 μL/min flow rate. Each sample was vortexed before data acquisition. 0.2μm filtered PBS was used as a negative control. The gating strategy was similar to the total bacterial density. The live, dying, and dead populations were identified in the BL1-H vs BL3-H scatterplot (**Supplementary Figure 5**).

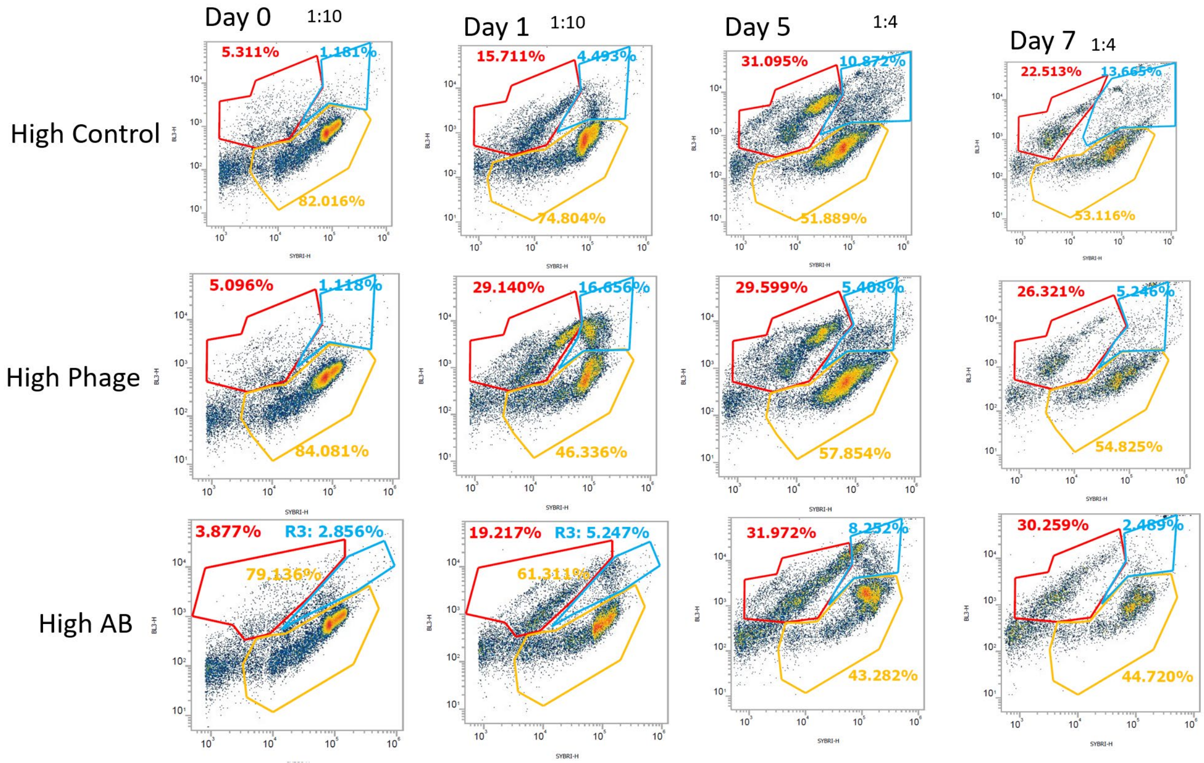

Supplementary Figure 5: *Example of live-, dying- and dead- population estimates in the microcosms added high amounts of F. columnare Fc7 at 0, 1, 5 and 7 DPI. The live-, dying- and dead- population was quantified in the BL1-H vs BL3-H scatterplot. Living = yellow gate, dying = blue, red = dead. Percentages indicate the proportion of events in the gate. The sample dilution factor is indicated above the plots. Note that both phage- and antibiotic treatment increased the proportion of dying and dead cells compared to control after 1 DPI.*

Statistical analysis workflow in R

Bacterial density polynomial model

To test for differences in bacterial density between the control and the treatments, we fitted a third-degree polynomial mixed effect model with the $\log_{10}$ transformed bacterial density as the response variable and treatment, sampling day, propagule pressure and the interactions between these as the explanatory variables. We used *lmer()* from the lme4 package (version 1.1-34) to create the model and *emmeans*() from the emmeans package (version 1.8.2-090007, github.com/rvlenth/emmeans) to obtain estimated marginal means for the density ratio difference between control and either phage- or antibiotic treatment. To estimate the conditional R^2 we used the *r.squaredGLMM*() function from the MuMIn package (version 1.47.5). P-values obtained from *emmeans*() were Dunnett adjusted within each sampling day, level of *F. columnare* Fc7 added and treatment comparison (n=42 comparisons). 95% confidence intervals were obtained from *emmip*() and used for plotting the temporal change in bacterial density.

Richness polynomial model

α-diversity was investigated as Hill diversity of order 0 (richness), 1 and 2 [3]. We first standardised the sample sequence depth by scaling to 26 448 reads using *transfom()* from the microbiome package (version 1.20.0, microbiome.github.io). This scaled dataset was rarefied using *rarefy_even_depth*() from phyloseq [4] (version 1.42.0) to ensure equal sampling depth. We used *renyi*() from vegan [5] (2.6-4) to estimate the α-diversity. We defined evenness to be order1/richness [6].

To test for differences in ASV richness between the control and the treatments, we fitted a second-degree mixed effect polynomial model with richness as the response variable and treatment, sampling day, level of *F. columnare* Fc7 added and the interactions between these as the explanatory variables using *lm()*. Sampling day was included as a random effect. As for bacterial density, *emmeans()* and *emmip()* were used to obtain the estimated marginal means.

Bacterial composition

Bacterial composition was evaluated based on both relative and absolute ASV abundances. To obtain absolute abundances, the ASV table was first transformed into compositional counts. Then each sample was scaled by the quantified bacterial density, and the ASV table was rounded to

the closest integer. Finally, bacterial composition bar plots were generated using functions from the package microshades (version 1.10, github.com/KarstensLab/microshades).

ASV1 was identified to be the added *F. columnare* Fc7. To test for statistical differences in the relative abundance of *F. columnare* Fc7 between treatment groups within each level of *F. columnare* Fc7 added, we used *kruskal.test*() and *pairwise.wilcox.test*() from the stats package.

Community composition comparisons

We estimated β-diversity using the Bray-Curtis and Sørensen similarity by averaging 100 similarity matrixes generated from randomly subsampled datasets using the function *avgdist()* from vegan [5, 7]. The datasets were subsampled to 26 448 reads. Principal coordinate analysis (PCoA) was used to visualise the variation in β-diversity and conducted using *ordinate*() and *plot_ordination*() from phyloseq. Within each sampling day and level of *F. columnare* Fc7 added, a permutational analysis of variance (PERMANOVA) with 999 permutations tested for differences in centroid location and dispersion between samples using the function *adonis2*() from vegan. Differences in group dispersions were tested using *betadisper*() from vegan. We performed 100 PERMANOVA and dispersion tests for each subset and averaged the test statistics.

To identify genera that changed in abundance due to the treatments, we performed differential abundance tests on samples taken at day 7. We used the packages corncob [8] (version 0.3.1), DESeq2 [9] (version 1.38.1) and ANCOMBC [10] (version 2.0.1) for differential abundance testing as different tools vary in which taxa are identified [11]. As input to all tools, we filtered the absolute abundance scaled ASV table to only contain ASVs with a total absolute abundance of over 2500 ASVs/mL and a prevalence of over 5%.

We used the function *differentialTest*() from corncob with the Wald significance test without bootstrapping separately for each propagule pressure (n=3 groups x 3 tests). For DeSeq2 and ANCOMB, the data were grouped according to treatment type and propagule pressure (n=9 groups). From DeSeq2, we used the function *DeSeq*() with parametric dispersion negative binomial GLM fitting and Wald significance testing on median ratio normalised count data. From ANCOMBC, we used the function *ancombc2*() with pairwise differential group testing and Holm p-value correction. We investigated the ASVs identified to be significant by all three methods. Because minor differences in abundance can be statistically, but maybe not biologically

significant, we focused on ASVs with over a 5-fold difference in absolute abundance between the control and the two treatments.

Community assembly

We quantified the change in replicate microcosm similarity per day to investigate the community assembly [12]. First, the Bray-Curtis and Sørensen similarity was quantified for each pair of replicate microcosms on each sampling day. Next, a mixed linear regression was performed with similarity as the response variable and sampling day, treatment type, amounts of added *F. columnare* Fc7 and the interaction between these as the explanatory variables. Sampling day was added as a random variable. Finally, the model's coefficient estimates and 95% confidence intervals were collected using *coefficient*() and *confint*() from stat.

Of particular interest was the temporal slope rate for each treatment. In the framework of Gundersen et al., 2021, the temporal slope is interpreted as a similarity rate. Positive similarity rates indicate that the community composition between two replicates became more similar over time, indicative of selection dominating community assembly. Negative rates, on the other hand, reflect that replicates became less similar over time, indicating that drift structured the community assembly. We quantified this similarity rate of change for each experimental group.

References:

1. Laanto E, Sundberg LR, Bamford JKH. Phage specificity of the freshwater fish pathogen Flavobacterium columnare. *Appl Environ Microbiol* 2011; 77: 7868–7872.
2. Glonti T, Pirnay J-P. In Vitro Techniques and Measurements of Phage Characteristics That Are Important for Phage Therapy Success. *Viruses 2022, Vol 14, Page 1490* 2022; 14: 1490.
3. Hill MO. Diversity and Evenness: A Unifying Notation and Its Consequences. *Ecology* 1973; 54: 427–432.
4. McMurdie PJ, Holmes S. phyloseq: An R Package for Reproducible Interactive Analysis and Graphics of Microbiome Census Data. *PLoS One* 2013; 8: e61217.
5. Oksanen J, Simpson G, Blanchet G, Kindt R, Legendre P, Minchin P, et al. vegan: Community Ecology Package. 2022.
6. Tuomisto H. An updated consumers guide to evenness and related indices. *Oikos* 2012; 121: 1203–1218.
7. Bray JR, Curtis JT. An Ordination of the Upland Forest Communities of Southern Wisconsin. *Ecol Monogr* 1957; 27: 325–349.
8. Martin BD, Witten D, Willis AD. Modeling microbial abundances and dysbiosis with beta-binomial regression. *https://doi.org/101214/19-AOAS1283* 2020; 14: 94–115.
9. Love MI, Huber W, Anders S. Moderated estimation of fold change and dispersion for RNA-seq data with DESeq2. *Genome Biol* 2014; 15: 550.
10. Lin H, Peddada S das. Analysis of compositions of microbiomes with bias correction. *Nature Communications 2020 11:1* 2020; 11: 1–11.
11. Nearing JT, Douglas GM, Hayes MG, MacDonald J, Desai DK, Allward N, et al. Microbiome differential abundance methods produce different results across 38 datasets. *Nature Communications 2022 13:1* 2022; 13: 1–16.
12. Gundersen MS, Morelan IA, Andersen T, Bakke I, Vadstein O. The effect of periodic disturbances and carrying capacity on the significance of selection and drift in complex bacterial communities. *ISME Communications* 2021; 1: 53.

Supplementary figures and tables

***Supplementary Table 1:** Coefficient estimates for the polynomial mixed effect model predicting the log_{10}-transformed bacterial density as a response to treatment, level of F. columnare Fc7 added and day. Sampling day was used as a random effects term. Model formula = log_{10}(density) ~ poly(day, degree = 3) * Treatment * Added Fc7 + (day|Sampling_unit). The control treatment and no added F. columnare Fc7 were set to intercept. Estimate = parameter estimate±standard error. t = t-value. Conditional R-squared: 0.780.*

Coefficient	Estimate±SE	t
(Intercept)	5.7±0.0	377.4
poly(day, degree = 3)1	0.2±0.3	0.7
poly(day, degree = 3)2	0.4±0.3	1.6
poly(day, degree = 3)3	0.3±0.3	1.0
TreatmentPhage	0.1±0.0	4.8
TreatmentAB	0.0±0.0	-2.2
Added_Fc7low	0.2±0.0	7.2
Added_Fc7high	0.3±0.0	14.7
poly(day, degree = 3)1:TreatmentPhage	1.4±0.4	3.5
poly(day, degree = 3)2:TreatmentPhage	0.1±0.4	0.3
poly(day, degree = 3)3:TreatmentPhage	-0.8±0.4	-2.0
poly(day, degree = 3)1:TreatmentAB	-0.2±0.4	-0.5
poly(day, degree = 3)2:TreatmentAB	0.2±0.4	0.6
poly(day, degree = 3)3:TreatmentAB	-1.1±0.4	-2.9
poly(day, degree = 3)1:Added_Fc7low	1.2±0.4	3.2
poly(day, degree = 3)2:Added_Fc7low	0.4±0.4	1.1
poly(day, degree = 3)3:Added_Fc7low	-1.0±0.4	-2.6
poly(day, degree = 3)1:Added_Fc7high	-2.1±0.4	-5.3
poly(day, degree = 3)2:Added_Fc7high	-1.1±0.4	-2.8
poly(day, degree = 3)3:Added_Fc7high	-2.8±0.4	-7.2
TreatmentPhage:Added_Fc7low	-0.1±0.0	-2.9
TreatmentAB:Added_Fc7low	-0.1±0.0	-2.9
TreatmentPhage:Added_Fc7high	-0.2±0.0	-6.0
TreatmentAB:Added_Fc7high	-0.1±0.0	-1.8
poly(day, degree = 3)1:TreatmentPhage:Added_Fc7low	-1.6±0.6	-2.9
poly(day, degree = 3)2:TreatmentPhage:Added_Fc7low	-0.1±0.5	-0.1
poly(day, degree = 3)3:TreatmentPhage:Added_Fc7low	1.1±0.5	2.1
poly(day, degree = 3)1:TreatmentAB:Added_Fc7low	-1.8±0.6	-3.3
poly(day, degree = 3)2:TreatmentAB:Added_Fc7low	-0.7±0.5	-1.3
poly(day, degree = 3)3:TreatmentAB:Added_Fc7low	0.9±0.5	1.7
poly(day, degree = 3)1:TreatmentPhage:Added_Fc7high	-1.3±0.6	-2.4
poly(day, degree = 3)2:TreatmentPhage:Added_Fc7high	-0.7±0.5	-1.2
poly(day, degree = 3)3:TreatmentPhage:Added_Fc7high	0.5±0.5	0.8
poly(day, degree = 3)1:TreatmentAB:Added_Fc7high	0.6±0.6	1.0
poly(day, degree = 3)2:TreatmentAB:Added_Fc7high	-0.4±0.5	-0.7
poly(day, degree = 3)3:TreatmentAB:Added_Fc7high	0.6±0.5	1.1

Supplementary Table 2: *The ratio between the estimated marginal mean in bacterial density for phage or antibiotic to the control treatment and the ratios standard error (SE), the degrees of freedom for each test (df), 95% confidence interval (lower-upper CI), the t-ratio value statistics, and the corresponding p-value (Student t-test based). P-values were Dunnett adjusted within each day and propagule pressure level (n=2 tests).*

	Day	Added Fc7	ratio	SE	df	Lower CI	Upper CI	t-ratio	p-value
Phage/none	0	no	1.13	0.15	126.08	0.85	1.52	0.97	0.53
Phage/none	1	no	0.98	0.08	63.37	0.81	1.18	-0.30	0.92
Phage/none	2	no	1.00	0.09	147.89	0.82	1.22	-0.02	1.00
Phage/none	3	no	1.14	0.09	151.44	0.96	1.36	1.66	0.18
Phage/none	4	no	1.35	0.10	145.06	1.14	1.61	3.94	<0.001
Phage/none	5	no	1.57	0.14	134.81	1.29	1.91	5.12	<0.001
Phage/none	6	no	1.66	0.14	59.43	1.37	2.02	5.91	<0.001
Phage/none	7	no	1.50	0.19	118.17	1.12	2.00	3.13	0.0044
Phage/none	0	low	1.03	0.14	119.49	0.77	1.39	0.26	0.94
Phage/none	1	low	1.12	0.09	58.71	0.92	1.35	1.32	0.33
Phage/none	2	low	1.11	0.10	143.65	0.92	1.35	1.24	0.37
Phage/none	3	low	1.06	0.08	145.05	0.90	1.25	0.77	0.65
Phage/none	4	low	1.00	0.08	139.29	0.84	1.18	-0.05	1.00
Phage/none	5	low	0.95	0.08	134.07	0.78	1.16	-0.54	0.80
Phage/none	6	low	0.96	0.08	59.36	0.79	1.17	-0.46	0.85
Phage/none	7	low	1.05	0.14	118.17	0.79	1.41	0.40	0.88
Phage/none	0	high	0.78	0.10	126.82	0.58	1.04	-1.91	0.11
Phage/none	1	high	0.79	0.06	58.85	0.65	0.94	-2.99	0.0080
Phage/none	2	high	0.82	0.07	140.98	0.68	1.00	-2.27	0.047
Phage/none	3	high	0.88	0.07	145.62	0.74	1.04	-1.76	0.15
Phage/none	4	high	0.92	0.07	146.27	0.77	1.09	-1.13	0.42
Phage/none	5	high	0.92	0.08	137.86	0.75	1.12	-0.96	0.53
Phage/none	6	high	0.86	0.07	60.35	0.70	1.04	-1.80	0.14
Phage/none	7	high	0.72	0.10	124.60	0.53	0.98	-2.40	0.034
AB/none	0	no	1.16	0.15	126.70	0.87	1.56	1.15	0.42
AB/none	1	no	0.83	0.07	59.53	0.69	1.00	-2.26	0.052
AB/none	2	no	0.76	0.07	144.32	0.63	0.93	-3.15	0.0039
AB/none	3	no	0.82	0.06	149.80	0.69	0.97	-2.68	0.0160
AB/none	4	no	0.92	0.07	146.62	0.78	1.10	-1.01	0.50
AB/none	5	no	1.01	0.09	137.69	0.83	1.24	0.16	0.97
AB/none	6	no	0.98	0.08	59.87	0.81	1.19	-0.23	0.95
AB/none	7	no	0.76	0.10	118.32	0.57	1.02	-2.12	0.0677
AB/none	0	low	1.02	0.13	126.48	0.76	1.36	0.12	0.98
AB/none	1	low	0.94	0.08	58.60	0.78	1.13	-0.80	0.64
AB/none	2	low	0.88	0.07	140.14	0.73	1.06	-1.54	0.22
AB/none	3	low	0.82	0.06	142.75	0.69	0.97	-2.66	0.0168
AB/none	4	low	0.75	0.06	139.29	0.64	0.89	-3.77	<0.001
AB/none	5	low	0.67	0.06	133.54	0.55	0.81	-4.62	<0.001
AB/none	6	low	0.56	0.05	59.04	0.46	0.68	-6.77	<0.001
AB/none	7	low	0.44	0.06	118.02	0.33	0.59	-6.39	<0.001
AB/none	0	high	0.77	0.11	128.82	0.56	1.04	-1.94	0.10
AB/none	1	high	0.71	0.06	59.78	0.59	0.86	-4.16	<0.001
AB/none	2	high	0.73	0.06	141.03	0.60	0.88	-3.78	<0.001
AB/none	3	high	0.78	0.06	146.27	0.66	0.92	-3.35	<0.001
AB/none	4	high	0.84	0.06	146.42	0.71	1.00	-2.25	0.050
AB/none	5	high	0.88	0.08	138.08	0.72	1.08	-1.38	0.29
AB/none	6	high	0.87	0.08	60.61	0.71	1.06	-1.64	0.19
AB/none	7	high	0.76	0.10	124.68	0.56	1.03	-2.01	0.087

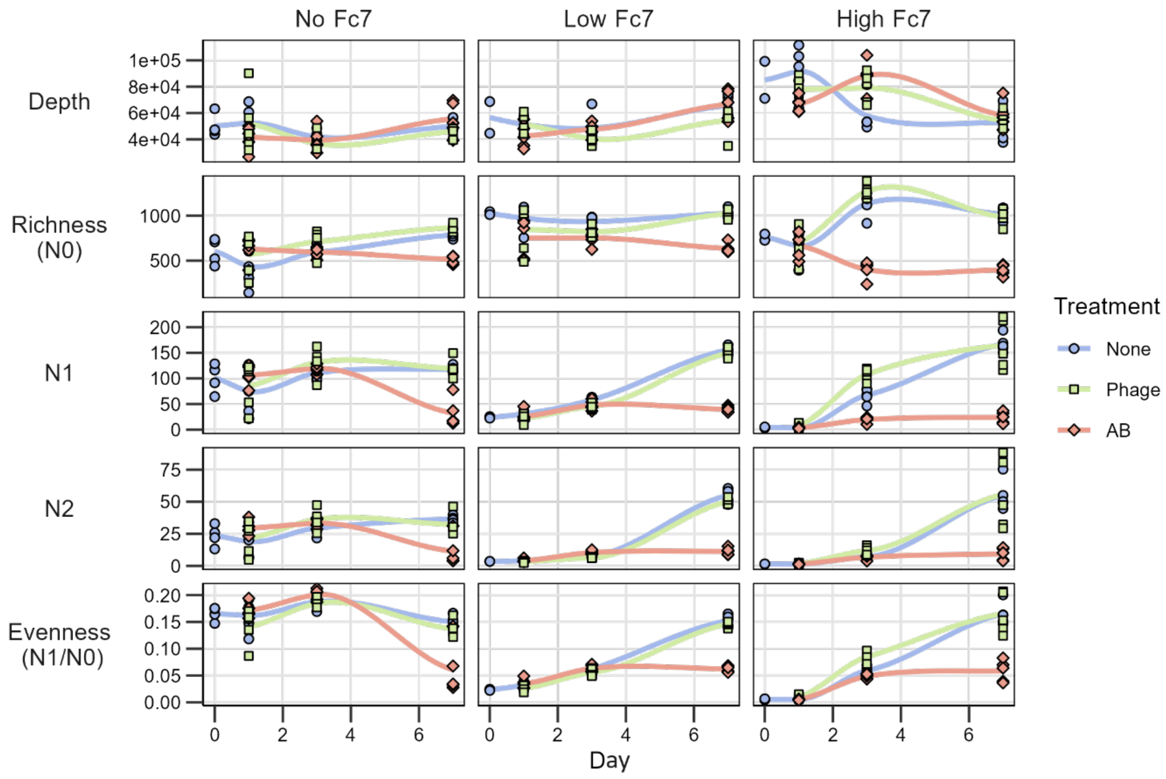

Supplementary Figure 6: *The sequencing depth, ASV richness (N_0), Hill-diversity of first (N_1) and second (N_2) order, and evenness (N_1/N_0) in each sample over time categorised according to level of F. columnare Fc7 added (none, low, high). The colours and shapes indicate the treatment type (none, phage treatment and antibiotics). The lines represent locally estimated scatterplot smoothing regression (loess).*

Supplementary Table 3: *Coefficient estimates ± standard error of the estimate and the t-test statistics associated with the polynomial mixed effect model for bacterial richness. Model formula = richness ~ poly(day, degree = 2) * Treatment * Added_Fc7 + (day|Sampling unit). The control treatment and no added F. columnare Fc7 were set to intercept. SE = standard error, t = t-value. Conditional R-squared: 0.796.*

Coefficient	Estimate±SE	t
(Intercept)	599.7± 35.6	16.8
poly(day, degree = 2)1	1667.3±406.7	4.1
poly(day, degree = 2)2	-128.5±311.6	-0.4
TreatmentPhage	108.7± 51.9	2.1
TreatmentAB	-19.1± 51.3	-0.4
Added_Fc7Low	376.8± 51.2	7.4
Added_Fc7High	342.4± 50.4	6.8
poly(day, degree = 2)1:TreatmentPhage	-164.8±595.7	-0.3
poly(day, degree = 2)2:TreatmentPhage	-189.1±536.5	-0.4
poly(day, degree = 2)1:TreatmentAB	-2230.8±595.7	-3.7
poly(day, degree = 2)2:TreatmentAB	83.8±508.2	0.2
poly(day, degree = 2)1:Added_Fc7Low	-1374.7±584.3	-2.4
poly(day, degree = 2)2:Added_Fc7Low	416.1±443.4	0.9
poly(day, degree = 2)1:Added_Fc7High	-438.2±575.2	-0.8
poly(day, degree = 2)2:Added_Fc7High	-1211.5±440.7	-2.7
TreatmentPhage: Added_Fc7Low	-188.3± 73.5	-2.6
TreatmentAB: Added_Fc7Low	-244.3± 73.1	-3.3
TreatmentPhage: Added_Fc7High	-97.5± 73.0	-1.3
TreatmentAB: Added_Fc7High	-419.4± 72.5	-5.8
poly(day, degree = 2)1:TreatmentPhage:Added_Fc7Low	765.8±848.7	0.9
poly(day, degree = 2)2:TreatmentPhage:Added_Fc7Low	412.4±740.6	0.6
poly(day, degree = 2)1:TreatmentAB:Added_Fc7Low	1349.4±848.7	1.6
poly(day, degree = 2)2:TreatmentAB:Added_Fc7Low	-613.5±720.3	-0.9
poly(day, degree = 2)1:TreatmentPhage:Added_Fc7High	313.3±842.4	0.4
poly(day, degree = 2)2:TreatmentPhage:Added_Fc7High	-1394.9±739.0	-1.9
poly(day, degree = 2)1:TreatmentAB:Added_Fc7High	-345.1±842.4	-0.4
poly(day, degree = 2)2:TreatmentAB:Added_Fc7High	2329.6±718.7	3.2

Supplementary Table 4: *The difference in estimated marginal means for ASV richness between the phage- or antibiotic treatment to the control and the standard error of the difference (SE), the degrees of freedom for each test (df), 95% confidence interval (lower-upper CI), the t-ratio value statistics, and the corresponding p-value (Student t-test based). P-values were Dunnett adjusted within each day and level of added F. columnare Fc7 (n=2 tests).*

contrast	Added Fc7	Day	Estimate difference	SE	df	Lower CI	Upper CI	t-ratio	p-value
**Phage - none**	no	1	113.49	86.65	40.62	-86.11	313.09	1.31	0.33
	low	1	-119.45	89.25	40.18	-325.11	86.22	-1.34	0.32
	high	1	-76.70	86.65	40.62	-276.30	122.91	-0.89	0.58
	no	3	133.66	85.13	76.66	-59.10	326.43	1.57	0.21
	low	3	-116.34	80.28	76.16	-298.16	65.48	-1.45	0.26
	high	3	193.65	80.14	76.22	12.16	375.13	2.42	0.0345
	no	7	81.71	74.44	41.80	-89.59	253.00	1.10	0.45
	low	7	-1.12	74.44	41.81	-172.43	170.18	-0.02	1.00
	high	7	-38.89	74.44	41.80	-210.19	132.40	-0.52	0.81
**AB - none**	no	1	172.09	86.65	40.62	-27.51	371.69	1.99	0.0995
	low	1	-214.65	89.25	40.18	-420.31	-8.98	-2.41	0.0397
	high	1	-107.50	86.65	40.62	-307.10	92.11	-1.24	0.3700
	no	3	10.46	80.14	76.22	-171.03	191.94	0.13	0.9800
	low	3	-185.94	80.28	76.16	-367.76	-4.12	-2.32	0.0442
	high	3	-675.15	80.14	76.22	-856.64	-493.67	-8.43	<0.001
	no	7	-271.89	74.44	41.80	-443.19	-100.60	-3.65	0.0014
	low	7	-387.12	74.44	41.81	-558.43	-215.82	-5.20	<0.001
	high	7	-632.29	74.44	41.80	-803.59	-461.00	-8.49	<0.001

Supplementary Table 5: *PERMANOVA test statistics and betadispersion p-value calculated for comparisons between phage- or antibiotic treatment to control at each sampling day within each level of F. columnare Fc7 added. For each comparison, 100 analyses were performed, and statistics were averaged. r2 = proportion of the total sum of squares, F = F-ratio of treatment effect, p = the P-value of the proportion of permuted pseudo-F-statistics greater than or equal to the observed statistic. A p-value < 0.05 indicates that the groups have significantly different centroid locations or dispersion. Beta p = P-value of betadisersion test evaluating H_0 = groups have the same dispersion around the centroid location. df = degrees of freedom for each PERMANOVA test.*

distance	comp	Day	Added_Fc7	r2	F	p	Permanova	df	Beta p	dispersion
Bray Curtis	None vs AB	1	no	0.20	2.00	0.054		9	0.056	
		1	low	0.12	1.00	0.430		8	0.250	
		1	high	0.04	0.31	0.980		9	0.570	
		3	no	0.17	1.60	0.180		9	0.570	
		3	low	0.73	22.00	0.009	SIG	9	0.009	different
		3	high	0.56	10.00	0.009	SIG	9	0.890	
		7	no	0.68	17.00	0.009	SIG	9	0.001	different
		7	low	0.84	42.00	0.009	SIG	9	0.040	different
		7	high	0.60	12.00	0.009	SIG	9	0.540	
	None vs Phage	1	no	0.07	0.57	0.790		9	0.650	
		1	low	0.16	1.40	0.180		8	0.630	
		1	high	0.32	3.70	0.073		9	0.033	different
		3	no	0.28	2.70	0.009	SIG	8	0.510	
		3	low	0.17	1.60	0.009	SIG	9	0.910	
		3	high	0.18	1.70	0.110		9	0.860	
		7	no	0.77	26.00	0.009	SIG	9	0.980	
		7	low	0.16	1.60	0.073		9	0.590	
		7	high	0.10	0.92	0.530		9	0.500	
Sørensen	None vs AB	1	no	0.14	1.30	0.210		9	0.220	
		1	low	0.14	1.20	0.200		8	0.170	
		1	high	0.09	0.82	0.930		9	0.970	
		3	no	0.16	1.50	0.072		9	0.550	
		3	low	0.43	5.90	0.009	SIG	9	0.170	
		3	high	0.57	11.00	0.009	SIG	9	0.001	different
		7	no	0.52	8.70	0.009	SIG	9	0.150	
		7	low	0.60	12.00	0.009	SIG	9	0.001	different
		7	high	0.66	15.00	0.009	SIG	9	0.001	different
	None vs Phage	1	no	0.10	0.90	0.430		9	0.490	
		1	low	0.11	0.90	0.500		8	0.380	
		1	high	0.13	1.20	0.140		9	0.960	
		3	no	0.25	2.40	0.009	SIG	8	0.520	
		3	low	0.12	1.10	0.100		9	0.270	
		3	high	0.15	1.40	0.009	SIG	9	0.097	
		7	no	0.26	2.80	0.009	SIG	9	0.009	different
		7	low	0.11	0.96	0.710		9	0.370	
		7	high	0.12	1.10	0.130		9	0.730	

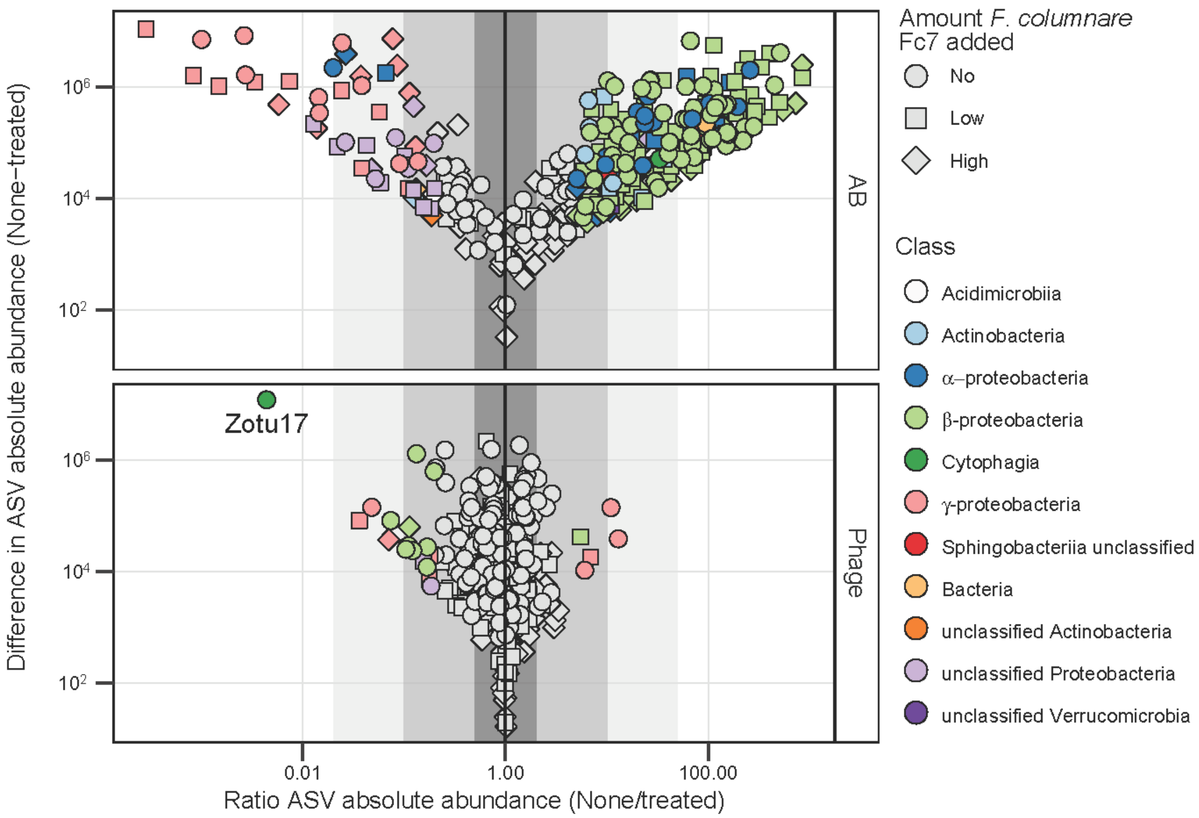

Supplementary Figure 7: *The average ratio and absolute difference in ASV absolute abundance between the control and treated microcosms. Note that the axes are log_{10} scaled. 126 ASVs were identified to be differentially abundant between control and either the phage- or antibiotic treatment at day 7. Each point represents a significant ASV detected in one of the nine groups. The ASV is coloured according to class if the ratio was over 5. The grey boxes highlight ratios of over 2, 10 and 50, going from dark grey to light grey. Abbreviations; Treatment: AB = antibiotics (Penicillin), N = no treatment, Phage = phage treatment (FCL-2). No Fc7 = uninvaded, Low or High Fc7 = 24% or 190% increase in density after addition of F. columnare Fc7.*

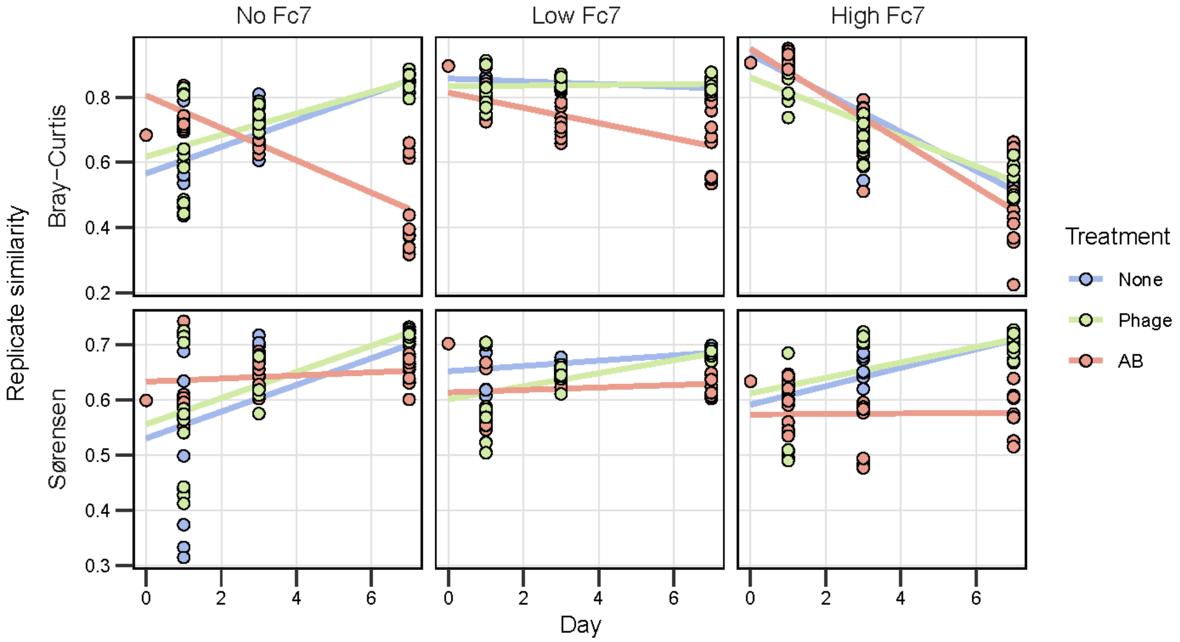

Supplementary Figure 8: *The Bray-Curtis (upper panels) and Sørensen (lower panels) similarity between replicate microcosms at each sampling day are indicated as points. The lines are linear regression performed for each group. All treatment groups within the same level of F. columnare Fc7 added have the same initial samples at day 0 (pre-treated samples).*
